# Depletion of DNA Polymerase Theta Inhibits Tumor Growth and Promotes Genome Instability through the cGAS-STING-ISG Pathway in Esophageal Squamous Cell Carcinoma

**DOI:** 10.3390/cancers13133204

**Published:** 2021-06-26

**Authors:** Jian Li, Josephine Mun-Yee Ko, Wei Dai, Valen Zhuoyou Yu, Hoi Yan Ng, Jean-Sébastien Hoffmann, Maria Li Lung

**Affiliations:** 1 Department of Clinical Oncology, University of Hong Kong, Hong Kong, China; james017@connect.hku.hk (J.L.); joko@hku.hk (J.M.-Y.K.); weidai2@hku.hk (W.D.); zvyu@hku.hk (V.Z.Y.); biancang@connect.hku.hk (H.Y.N.); 2 Laboratoire d’Excellence Toulouse Cancer (TOUCAN), Laboratoire de Pathologie, Institut Universitaire du Cancer-Toulouse, 31037 Toulouse, France; jean-sebastien.hoffmann@inserm.fr

**Keywords:** POLQ, genomic instability, innate immune response

## Abstract

**Simple Summary:**

DNA polymerase theta, encoded by the human *POLQ* gene, is upregulated in several cancers and is associated with poor clinical outcomes. The importance of *POLQ*, however, has yet to be elucidated in esophageal cancer. In this study, we explored the functional impacts of *POLQ* and looked into its underlying mechanisms. *POLQ* was overexpressed in esophageal squamous cell carcinoma (ESCC) tumors associated with unfavorable prognosis and contributed to malignant phenotypes by promoting genome stability, suggesting that targeting polymerase theta may provide a potential therapeutic approach for improving ESCC management.

**Abstract:**

Overexpression of the specialized DNA polymerase theta (POLQ) is frequent in breast, colon and lung cancers and has been correlated with unfavorable clinical outcomes. Here, we aimed to determine the importance and functional role of POLQ in esophageal squamous cell carcinoma (ESCC). Integrated analysis of four RNA-seq datasets showed *POLQ* was predominantly upregulated in ESCC tumors. High expression of *POLQ* was also observed in a cohort of 25 Hong Kong ESCC patients and negatively correlated with ESCC patient survival. *POLQ* knockout (KO) ESCC cells were sensitized to multiple genotoxic agents. Both rH2AX foci staining and the comet assay indicated a higher level of genomic instability in *POLQ*-depleted cells. Double KO of *POLQ* and *FANCD2*, known to promote POLQ recruitment at sites of damage, significantly impaired cell proliferation both in vitro and in vivo, as compared to either single *POLQ* or *FANCD2* KOs. A significantly increased number of micronuclei was observed in *POLQ* and/or *FANCD2* KO ESCC cells. Loss of *POLQ* and/or *FANCD2* also resulted in the activation of cGAS and upregulation of interferon-stimulated genes (ISGs). Our results suggest that high abundance of *POLQ* in ESCC contributes to the malignant phenotype through genome instability and activation of the cGAS pathway.

## 1. Introduction

Esophageal cancer (EC) was the eighth most frequent cancer and the sixth most prevalent cause of the cancer-related mortality worldwide in 2020 [1]. Esophageal squamous cell carcinoma (ESCC) is the main histological subtype, accounting for about 90% of ECs [2]. The majority of ESCC cases present only subtle, if any, symptoms until the late stages, which results in delayed diagnosis and unfavorable clinical outcomes [3]. Therefore, it is critical to identify novel clinical biomarkers and druggable targets for the better management of ESCC.

DNA polymerase theta (POLQ), encoded by the *POLQ* gene in humans, is an error-prone A-family DNA polymerase involved in several biological activities including DNA double-strand break (DSB) repair, translesion synthesis, base-excision repair (BER), and the repair of replication forks [4,5,6,7]. It is a critical component of alternative end joining (alt-EJ), which acts as the predominant DSB repair in mammalian cancer cells under circumstances of defective homology-directed repair [8]. The expression of *POLQ* is normally repressed in somatic cells but upregulated in several human cancers. It was firstly found in a Japanese study that *POLQ* was upregulated in tumor tissues, as compared with the paired non-tumor control samples in lung, colon and gastric malignancies [9]. A French study of colorectal cancer revealed that *POLQ* was among a list of 47 DNA replication-related genes, whose overexpression in tumors was significantly associated with poorer patient survival [10]. *POLQ* was also frequently upregulated in a group of oral squamous cell carcinomas from Brazil [11]. In addition, *POLQ* levels were found to be remarkably high in homologous recombination (HR)-deficient breast and ovarian cancers and correlated with unfavorable clinical outcomes [8,12].

Despite the important roles that *POLQ* plays in various cancers, there have been few studies characterizing its functional role in esophageal cancers to date. Our previous study utilizing the targeted gene next-generation sequencing (NGS) approach revealed the germline loss-of-function (LOF) mutations of *POLQ* and other DNA damage repair-related genes (*BRCA2* and *MSH2*) associated with the elevated risk of familial ESCC [13]. Nevertheless, the expression level of wildtype *POLQ* and its functions in ESCC have yet to be reported. In this study, we investigated the expression level and the functional impact of *POLQ* in ESCC to understand its mechanistic function.

Several recent studies have associated the deficiency of DNA damage repair genes (such as *BRCA2*) with the activation of innate immunity through the cGAS-STING pathway [14,15,16,17,18]. The 2’,3’-cGAMP, produced by cyclic GMP-AMP (cGAMP) synthase (cGAS) upon sensing the aberrant or self-leaked cytosolic DNA, activates the stimulator of interferon genes (STING) proteins and this leads to the expression of Type I interferon (IFN) and the secretion of other cytokines and chemokines triggering the anti-cancer immune response [19,20,21,22]. In this study, we also explored the potential innate immunity activation upon the loss of *POLQ* via the cGAS-STING pathway.

## 2. Materials and Methods

### 2.1. Clinical Specimens

Twenty-five pairs of ESCC patient tissues were collected from Hong Kong Queen Mary Hospital between 2001 and 2003, as previously reported [23]. Approval for this study was obtained from the Hospital Institutional Review Board at the University of Hong Kong (IRB UW-14-457).

### 2.2. RNA Sequence Analysis

We sequenced the RNAs of four tumor and non-tumor pairs of patient tissues using the Illumina HiSeq 2000 (San Diego, CA, USA) (2 × 100 bp paired reads). Three sets of public RNA sequencing (RNA-seq) data (SRP007169, SRP008496, SRP064894) were obtained from Sequence Read Archive (SRA) database. All the clean RNA-seq reads were aligned to reference genome hg19 using TopHat (version 2.0.14, bowtie version 2.2.4, College Park, MD, USA) [24]. The gene expression levels (FPKM) were calculated using Cufflinks (version 2.2.1, Seattle, WA, USA) [25].

### 2.3. Cell Lines

The immortalized human normal esophageal epithelial cell line NE1, human embryonic kidney 293T cell line, human colon cancer cell line RKO, human osteosarcoma cell line U2OS and 13 ESCC cell lines used in this study were cultured as previously described [12,26,27]. KYSE70TS and KYSE180TS were derived from subcutaneous nude mouse tumors established with KYSE70 and KYSE180 cell lines, respectively. Cell line authentication by STR DNA profiling was performed for all cell lines used. Cell lines were tested routinely for mycoplasma contamination with both 4’,6-diamidino-2-phenylindole staining and polymerase chain reaction amplification of DNA [26].

### 2.4. Plasmids and Lentivirus Preparation and Infection

Clustered Regularly Interspaced Short Palindromic Repeat (CRISPR) systems were used with sgRNA oligos (sequences of oligos are listed in the Table S1) targeting *POLQ* and *FANCD2* to generate *POLQ* and *FANCD2* knockout (KO) cell lines, respectively [27]. Lentivirus preparation and infection were performed as described [28]. To establish the *POLQ/FANCD2* double KO cell lines, ESCC cells were co-incubated with a pool of lentiviruses containing 2 KO oligos for *POLQ* and 2 KO oligos for *FANCD2* in the presence of 5mg/mL polybrene. The non-targeting oligo with a sequence of GTTCCGCGTTACATAACTTA was used as a CRISPR negative control [29].

### 2.5. RNA Isolation and Real-Time Quantitative Polymerase Chain Reaction

RNA isolation, reverse transcription, and quantitative polymerase chain reaction (Q-PCR) were performed as described [30]. Q-PCR was used to determine gene expression at the mRNA level in cell lines and tissue samples. FastStart™ Universal SYBR Green Master (Rox) (Roche Applied Science, Basel, Switzerland) was used according to the manufacturer’s instructions. Human glyceraldehyde 3-phosphate dehydrogenase (GAPDH) and mouse TATA-box-binding protein (TBP) were used as the endogenous loading controls for human and mouse genes, respectively. All Q-PCR reactions were carried out with the LightCycler 480 System (Roche, Basel, Switzerland) using the default SYBR green protocol. The expression level of the target gene was compared with the reference gene by calculating their fold differences using the 2^−ΔΔCt^ method. Experiments were repeated 3 times independently. Expression with average fold-changes larger than 2 or smaller than 0.5 were considered altered. All primers used in this study for Q-PCR are listed in Table S2.

### 2.6. Protein Extraction and Western Blot Analysis

Protein extraction was performed, as previously described [31]. Cell protein lysates were electrophoresed on 6% SDS-PAGE gels for POLQ analysis and 10% SDS-PAGE gels for all other protein targets. Proteins were transferred to PVDF membranes, blocked with 3% bovine serum albumin (BSA) and incubated with primary antibodies, as previously described [28]. POLQ antibodies are mouse monoclonal antibodies, affinity purified with protein A ceramic hyperDF (Akta System, Marlborough, MA, USA) (0.1 mg/mL). They were raised against the peptide antigens CSIFRARKRASLDINKEKPG, derived from regions of the central domain of POLQ. Their specificity was checked using an siRNA strategy [32]. Detailed information for the antibodies used in this study is summarized in Table S3.

### 2.7. MTT Assay Following Genotoxic Drug Treatments

The proliferation and viability of cells were determined by the 3-(4,5-dimethylthiazol-2-yl)-2,5-diphenyltetrazolium bromide (MTT) assay, as previously described [23]. Cells were counted and seeded at a concentration of 3 × 10^3^ cells per well in triplicate in 96-well cell culture microplates. Fresh medium with 10% fetal bovine serum and stated concentrations of the designated drug was added on day 2. Measurements were taken starting from day 3, as described earlier [28]. As dimethyl sulfoxide (DMSO) was used for the drug dilution, medium containing equivalently diluted DMSO was added to control cells.

### 2.8. Colony Formation Assay

Cells were seeded in 12-well plates at a concentration of 5 × 10^3^ cells/well. After a 10-day culture, cells were fixed in 4% paraformaldehyde followed by 1 × Giemsa stain (Sigma Aldrich, Saint Louis, MO, USA). Excess Giemsa was removed by rinsing the plate with running water. Images were then captured and cell colonies were counted using the Gel Doc XR system (Bio-Rad Laboratories, Hercules, CA, USA).

### 2.9. Ionizing Radiation

Ionizing radiation was used to induce different degrees of DNA DSBs in living cells. A cell irradiator with cesium as its radioactive source, MDS Gammacell 3000 Elan II (Nordion, Ottawa, ON, Canada), was operated by well-trained and licensed individuals rigorously following the manufacturer’s manual.

### 2.10. Immunofluorescence (IF) Staining and Confocal Microscopy

Cells were seeded on pre-sterilized 22 mm square coverslips placed in 35 mm plates and cultured for 1–2 days until roughly 60% confluence was reached. On the day of staining, cells were gently washed with PBS and fixed in 4% paraformaldehyde (PFA) in PBS at room temperature for 15 min. After washing with PBS three times, cells were subjected to permeabilization by incubating with 0.3% Triton-X100 in PBS at room temperature for 20 min. Cells were then washed three times with PBS and blocked with 4% BSA in TBST for 60 min at room temperature. Upon the removal of the blocking buffer, cells were incubated with diluted primary antibody overnight at 4 °C. On the next day, cells were rinsed twice with PBS and then incubated with Alexa Fluor-conjugated antibodies (Invitrogen, Waltham, MA, USA) at room temperature for 90 min, avoiding light exposure. The coverslips were washed three times with PBS and then mounted onto the slides using SlowFade™ Gold Antifade Mountant (Thermo Fisher Scientific, Waltham, MA, USA). Images were captured by LSM800 confocal microscopy (Zeiss, Jena, Germany) or BX51 fluorescence microscopy (Olympus, Tokyo, Japan).

### 2.11. Single Cell Gel Electrophoresis Assay (Comet Assay)

The alkaline comet assay was performed according to a method published in *Nature Protocol* [33] with only minor optimizations. In brief, around 1 × 10^4^ cells were collected and resuspended in 100 µL 0.6% UltraPure™ (Thermo Fisher Scientific, Waltham, MA, USA) low melting agarose dissolved in PBS. Then 50 µL of the suspension was cast onto microscope slides precoated with 1% normal melting agarose. Another layer of 0.6% low melting agarose was then added on top before cells were lysed overnight at 4 °C in lysis buffer (2.5 M NaCl, 0.1 M EDTA, 10 mM Trizma Base, 1% N-laurylsarcosine, 10% DMSO, pH = 10, freshly supplied with 0.1% Triton X-100 before use). On the next day, electrophoresis was conducted at 4 °C for 60 min at 15 V in electrophoresis buffer (300 mM sodium acetate, 100 mM Tris-HCL, pH = 8.3). The slides were then rinsed with PBS, dehydrated/fixed using absolute ethanol, and stained with 5 μg/mL DAPI before mounting with coverslips. Images were captured with BX51 fluorescence microscopy (Olympus, Tokyo, Japan) and analyzed with OpenComet software (Cambridge, MA, USA) [34].

### 2.12. Enzyme-Linked Immunosorbent Assay (ELISA)

To measure the level of CCL5 protein secreted by the cells, the Human CCL5/RANTES Quantikine ELISA Kit (R&D Systems, Minneapolis, MN, USA) was used according to the manufacturer’s protocol. For in vitro cultured cells, conditioned medium was collected two days after the cells were seeded in a 6-well microplate (Thermo Fisher Scientific, Waltham, MA, USA). The conditioned medium containing the proteins was first spun down to remove the cell debris and then concentrated using a 3 K MWCO Microsep™ Advance Centrifugal Device (Pall, New York, NY, USA). For mouse subcutaneous tumors, tissues were first homogenized in radioimmunoprecipitation buffer (100 mM of sodium chloride, 1% of Triton X-100, 0.5% of sodium deoxycholate, 0.1% of SDS, in 50 mM of Tris-Cl buffer, pH = 7.4) at 4 °C before centrifugation for the removal of the insoluble components. The BSA assay was performed to standardize the protein concentrations in each sample. The absorbance was measured at a wavelength of 450 nm using the ELX800 Absorbance Microplate Reader (BioTek, Winooski, VT, USA) and the readings were corrected by subtracting the reading at 540 nm to achieve higher accuracy.

### 2.13. In Vivo Tumorigenicity Assay

Female BALB/c/nu/nu athymic nude mice (7–9 weeks of age) were obtained from and housed in the Laboratory Animal Unit of the University of Hong Kong. The housing environment was kept between 16 °C and 26 °C with relative humidity between 30–70% under a regular 12-h light, 12-h dark cycle. All experimental procedures were approved by the Committee on the Use of Live Animals in Teaching and Research in the University of Hong Kong. Mice were randomly assigned to different cell line groups with three mice in each group. An optimal number (1.8 × 10^6^/site for SLMT and 2.4 × 10^6^/site for KYSE180TS) of cells resuspended in serum-free medium and injected subcutaneously into both flanks of the mice. The mouse’s health condition was monitored closely and the subcutaneous tumor volume was measured weekly using a caliper. Cervical dislocation was used for animal euthanasia.

### 2.14. Statistical Analysis

Data are presented as the mean ± SD. Two-sided Student’s *t*-test was applied unless stated otherwise. The results were considered as statistically significant when the *p* value was less than 0.05.

## 3. Results

### 3.1. POLQ Is Upregulated in ESCC and Correlates with Unfavorable Clinical Outcome

**Figure 1:**
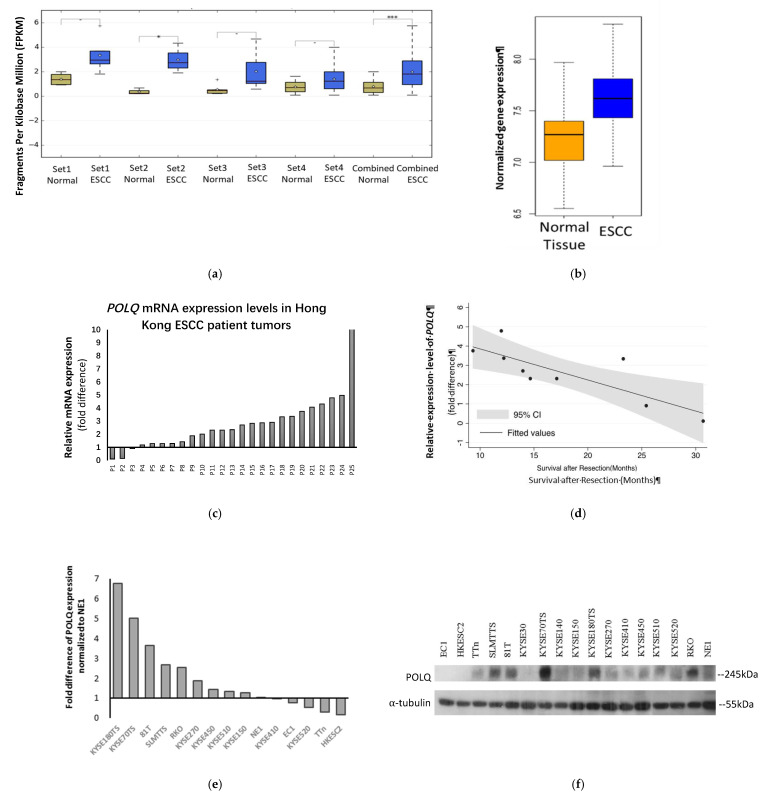
*POLQ* is upregulated in ESCC and correlates with unfavorable clinical outcome: (**a**) RNA-seq transcriptome from one in-house and three NCBI SRA datasets (accession numbers are SRP007169, SRP008496, and SRP064894): set 1, *n* = 5 pairs Hong Kong ESCC samples (in-house); set 2, *n* = 3 pairs; set 3, 5 normal and 7 ESCC tumors, set 4, *n* = 15 pairs (* *p* < 0.05, *** *p* < 0.001). (**b**) NCBI GEO microarray dataset: GSE23400, *n* = 53. (**c**) Relative *POLQ* mRNA expression levels in Hong Kong ESCC patient tumors. (**d**) Relative *POLQ* mRNA expression levels correlated with survival after resection for ESCC-related deaths: *n* = 9 (simple regression analysis, R^2^ = 0.656, *p* = 0.008). *POLQ* expression in ESCC cell lines at the mRNA and protein levels were determined by Q-PCR (**e**) and Western blotting (**f**), respectively.

To determine the clinical significance of *POLQ* in ESCC, the *POLQ* expression was first examined in paired ESCC tumors and adjacent normal tissues using RNA-seq. In our transcriptomic profiling analysis, *POLQ* was found to be overexpressed in ESCC tumors, when compared with the adjacent normal esophageal epithelial tissues in all three public and one in-house RNA-seq datasets (Figure 1a). In line with this result, *POLQ* upregulation in ESCC tumor was found in a NCBI GEO microarray dataset (GSE23400) (Figure 1b). We then determined the *POLQ* mRNA levels in 25 Hong Kong ESCC patient tumors and paired adjacent normal tissues using Q-PCR. As shown in Figure 1c, *POLQ* was upregulated in 16 out of 25 of these ESCC tumor-normal pairs. When stratifying these 25 ESCC patients by the cause of death, we found 9 patients died due to ESCC, while others died of other unrelated causes. By applying the simple regression analysis model, a negative correlation was found between the relative mRNA expression levels of *POLQ* of these 9 patients and their survival times after surgical resection. As shown in Figure 1d, the high expression of *POLQ* in ESCC tumors was associated with unfavorable survival time after resection (R^2^ = 0.656, *p* = 0.008).

Consistently, the expression of the *POLQ*, at both mRNA and protein levels, was upregulated in 62% (8/13) of ESCC cell lines using an immortalized normal esophageal epithelial cell line, NE1, as a reference (Figure 1e,f). Four ESCC cell lines, namely, KYSE70TS, KYSE180TS, 81T and SLMT, had higher *POLQ* expression levels even when compared with RKO, a colon cancer cell line well-known for expressing high endogenous levels of *POLQ*.

### 3.2. POLQ Maintains Genome Stability in ESCC Cells

To better characterize the functional impacts of *POLQ* in ESCC, *POLQ* was knocked out using the CRISPR technology in high-*POLQ* expressing ESCC cell lines for further investigation. The successful depletion of POLQ protein in ESCC cell lines was validated by Western blotting (Figure S1).

**Figure 2:**
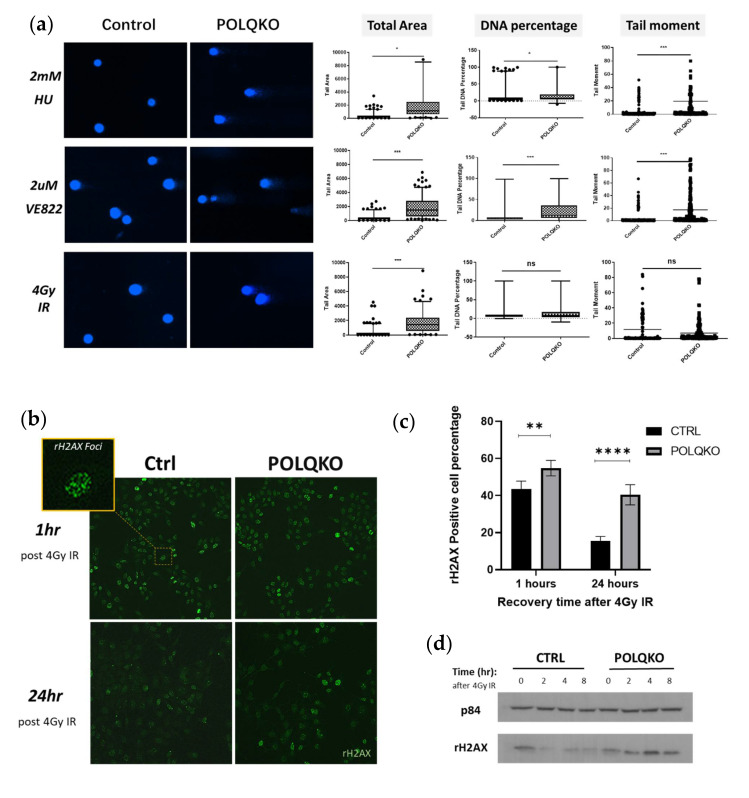

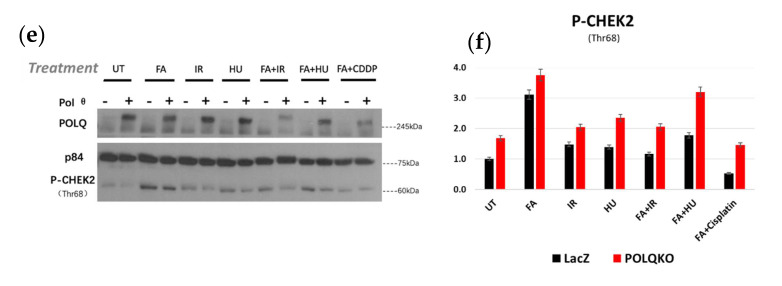
*POLQ* maintains genome stability in ESCC cells. (**a**) *POLQ* KO impaired the DNA damage repair efficiency in ESCC cells when exposed to DNA damage or replication stress inducers (Student’s t-test, *, *p* < 0.05, ***, *p* < 0.001). Error bars represent the ± SD. (**b**) Representative images for rH2AX foci formation assay. Magnification: 20×/40× (zoomed in image). (**c**) Quantification of the staining results (cells with 10 or more foci were defined as the positive cells, Student’s t-test, **, *p* < 0.01; ****, *p* < 0.0001). Data are presented as the mean ± SD. (**d**) The phosphorylation levels of CHK2 in untreated/treated KYSE180SE cells depleted or not for *POLQ* were measured by Western blotting (**e**) and quantified by ImageJ software (Bethesda, MD, USA) in (**f**) UT, untreated; FA, 1 mM formaldehyde; 4Gy ionizing radiation (IR); HU, 4 mM hydroxyurea; CDDP, 2 uM cisplatin; LacZ, negative control of CRISPR KO; p84, loading control. Data are presented as the mean ± SD.

We first compared the DNA damage repair efficiency of POLQ-depleted versus control ESCC cells exposed to different treatments by monitoring DNA breaks remaining after incubation to allow repair to proceed with a single cell gel electrophoresis approach, the alkaline comet assay [35]. Three different parameters, namely, tail area, tail DNA percentage and tail moment were used for the quantification. Significant higher levels of DNA damage assessed by all parameters were found in POLQ-depleted cells, as compared with the control cells, when treated with 2 mM replication stress inducer hydroxyurea or 2 µM ATR inhibitor VE822 (Figure 2a). Meanwhile, the POLQ depletion group exhibited a higher level of DNA damage, as compared with the control group, upon 4Gy ionizing radiation treatment, by using the tail area as the parameter (*p* < 0.001) (Figure 2a). This difference, however, was not statistically significant when analyzed using tail DNA percentage or tail moment as the parameter (Figure 2a). Therefore, we used an additional method to visualize and quantify DNA damage (especially DNA DSBs) after IR, the γH2AX foci formation assay [36]. Control and *POLQ* KO ESCC cells were treated with 4Gy radiation and the γH2AX foci formation assay was performed after 1 and 24 h post treatment (Figure 2b,c). Cells with 10 or more γH2AX foci were defined as the positive cells. A slightly higher percentage of γH2AX positive cells was observed in the *POLQ* KO group than in the control group one hour after the radiation treatment. After 24 h of recovery, there were still more than 40% γH2AX foci-positive cells in the *POLQ* KO group, while only less than 20% of the cells in the control group were γH2AX positive (Figure 2b,c). Basal levels of DNA damage in control and POLQ-depleted cells were measured without IR treatment (Figure S2). Delayed DSB repair upon POLQ depletion was also validated by Western blotting probing γH2AX (Figure 2d). Collectively, these results indicate that depleting POLQ significantly affects DNA break repair in ESCC.

Western blotting was next performed to assess the activation of DNA damage checkpoint proteins upon the POLQ depletion. In the absence of external stress, POLQ loss resulted in endogenous enhanced phosphorylation of checkpoint kinase 2 (CHEK2) at the site of Thr68 (Figure 2e,f). When exposed to single/dual replication stress/DNA damage inducers, CHEK2 activation seen in control cells was further exacerbated when POLQ was depleted (Figure 2e,f). No significant change was found in ATM, ATR, or CHEK1 phosphorylation status upon POLQ depletion (Figure S3). Taken together with the findings of undermined DNA damage repair efficiency in POLQ-depleted cells, these results suggest a protective role of *POLQ* in maintaining the genome stability of ESCC cells.

### 3.3. POLQ Depletion Sensitizes ESCC Cells to Mutiple Genotoxic Agents

**Figure 3:**
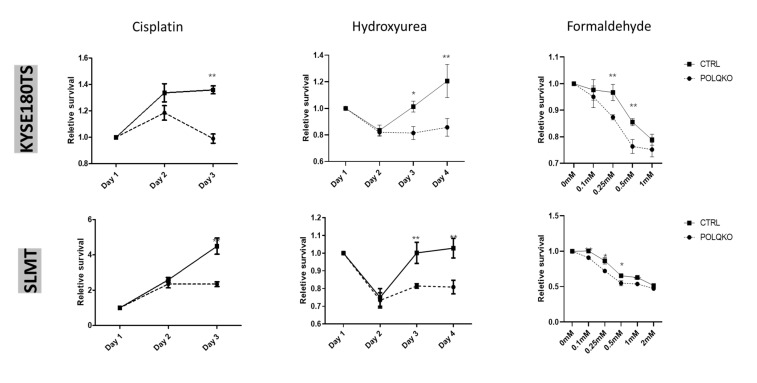

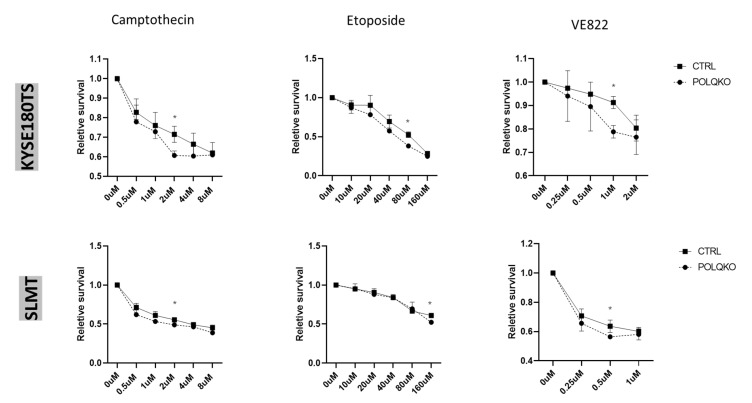
*POLQ* depletion sensitizes ESCC cells to multiple genotoxic agents. Cisplatin, hydroxyurea, formaldehyde, camptothecin, etoposide and VE822 were used to treat KYSE180TS and SLMT cell lines. Relative survival curves are illustrated. Student’s *t*-test, * *p* < 0.05; ** *p* < 0.01. Data are presented as the mean ± SD.

To determine whether *POLQ* may promote the genome stability of ESCC cells, we examined the impact of POLQ depletion on cell viability. POLQ-depleted and control ESCC cells were treated with different genotoxic agents including conventional platinum-based chemotherapy drug cisplatin, replication stress inducer hydroxyurea, environmental toxin formaldehyde, topoisomerase inhibitors camptothecin and etoposide and ATR inhibitor VE822 before being subjected to the MTT assay. Compared with the control cells, *POLQ* KO ESCC cells were, to different extents, sensitized to all these cytotoxic drugs (Figure 3). Consistently, these data indicate that *POLQ* expression favors maintenance of ESCC genome stability and cell viability.

### 3.4. Double Knockout POLQ and FANCD2 Drastically Inhibits ESCC Growth Both In Vivo and In Vitro

**Figure 4:**
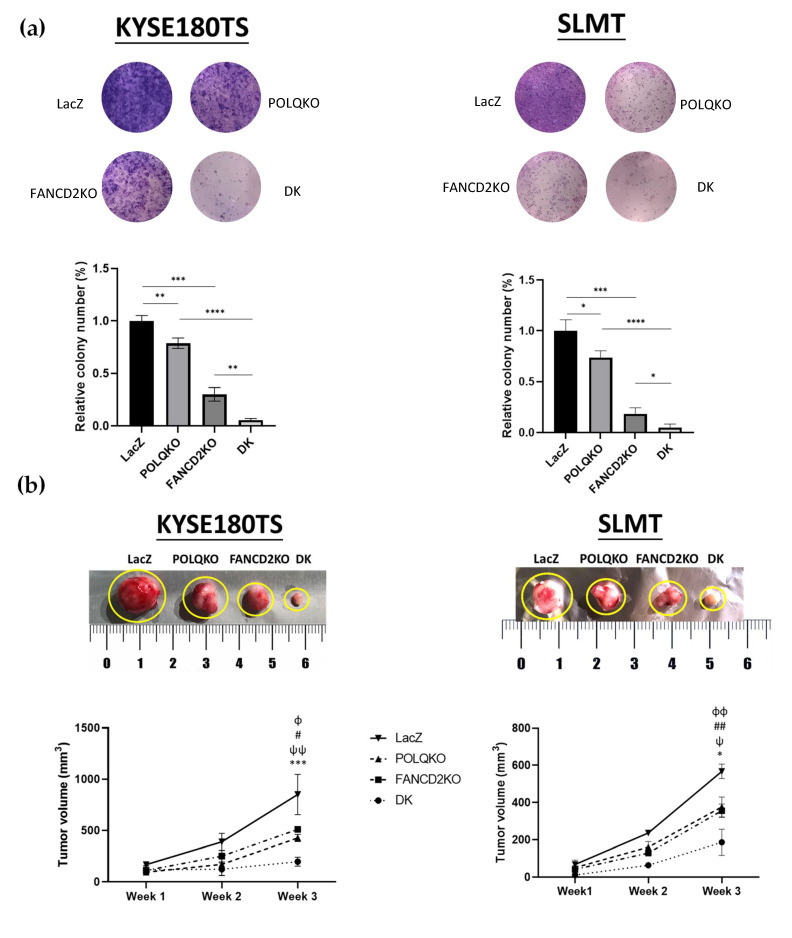
Double KO *POLQ* and *FANCD2* drastically inhibits ESCC growth both in vivo and *in vitro*. (**a**) *POLQ/FANCD2* double KO significantly inhibited ESCC cell colony formation in comparison to the single gene KOs. Experiments were done in triplicates in three independent experiments. Student’s *t*-test, *, *p* < 0.05; **, *p* < 0.01; ***, *p* < 0.001; ****, *p* < 0.0001. Data are presented as the mean ± SD. (**b**) *POLQ/FANCD2* double KO significantly inhibited the tumorigenicity of ESCC cell lines vs single gene KOs and control in vivo (*n* = 6; Student’s *t*-test, KYSE180TS, ɸ, LacZ vs. POLQKO, *p* < 0.05; #, LacZ vs. FANCD2KO, *p* < 0.05; ψψ, POLQKO vs. DK, *p* < 0.01; ***, FANCD2KO vs. DK, *p* < 0.001; SLMT, ɸɸ, LacZ vs. POLQKO, *p* < 0.01; ##, LacZ vs. FANCD2KO, *p* < 0.01; ψ, POLQKO vs. DK, *p* < 0.05; *, FANCD2KO vs. DK, *p* < 0.05). The tumor samples were collected at the end of week three. Data are presented as the mean ± SD.

While POLQ depletion strongly affected ESSC cell viability under treatment with genotoxic agents, low impact on cellular proliferation was observed under normal growth conditions (Figure 4), suggesting that the majority of endogenous DNA damage in ESCC cells can be repaired in the absence of *POLQ*. *FANCD2*, another DNA damage repair gene upregulated in ESCC [37], functions in both the Fanconi anemia pathway that repairs inter-strand DNA crosslinks and the homologous recombination (HR) pathway that repairs DNA DSBs [8,38,39]. FANCD2 has also been proven to be a central player in orchestrating DNA repair pathway choice at the replication fork and promotes POLQ recruitment at sites of damage [40]. Interestingly, our previous ESCC functional study demonstrating that *FANCD2* depletion significantly inhibited tumor growth and metastasis, already showing that DNA damage repair is essential for ESCC progression [30]. Since both *POLQ* and *FANCD2* are DNA damage repair genes conferring tumor progression, we explored the possible synergistic or synthetic lethality relationships between *POLQ* and *FANCD2* in ESCC tumorigenesis.

To compare the functional impact of *POLQ* single KO, *FANCD2* single KO, *POLQ*/*FANCD2* double KO and the control ESCC cells, we constructed the *FANCD2* KO (FANCD2KO) and *POLQ*/*FANCD2* KO (DK) ESCC cell lines. FANCD2KO cell lines were established as previously described [37]. DK cell lines were established by infecting the ESCC cells with a mixture of lentiviruses carrying two KO oligos for *POLQ* and two KO oligos for *FANCD2*. Successful KOs were validated by Western blotting (Figure S4).

The 2D colony formation assay was conducted to evaluate clonogenic ability in *POLQ/FANCD2* single/double KO ESCC cells. Both *FANCD2* single KO and *POLQ* single KO ESCC cells formed significantly fewer colonies than the control cells, whereas *POLQ/FANCD2* double KO cells had less colonies than with the single KOs (Figure 4a). The clonogenic ability was reduced by 22% in the *POLQ* KO group, 69% in *FANCD2* KO group and 94.5% in *POLQ*/*FANCD2* double KO group in the KYSE180TS cell line (Figure 4a). Similar results were also found with the SLMT cell line, in which the clonogenic ability was reduced by 26% in the *POLQ* KO group, 78% in the *FANCD2* KO group and 95% in *POLQ*/*FANCD2* double KO group (Figure 4a).

To examine the impact of the *POLQ/FANCD2* double KO on ESCC cell proliferation in vivo, the subcutaneous tumorigenicity assay was conducted on *BALB/c* nude mice. *POLQ/FANCD2* single/double KO and control KYSE180TS/SLMT cells were injected into both flanks of the mice and the tumor volumes were measured weekly for 3 consecutive weeks. In both cell lines tested, the *POLQ* or *FANCD2* single KO group had significantly smaller tumors in contrast to the control group, whereas the *POLQ*/*FANCD2* double KO group resulted in the drastic synergistic inhibition of subcutaneous tumors compared to the two single KO groups (Figure 4b). These in vitro and in vivo findings collectively suggest a potential synthetic lethality relationship between *POLQ* and *FANCD2* in ESCC.

### 3.5. Double Knockout of POLQ and FANCD2 Significantly Induces Genome Instability and the Formation of Micronuclei

**Figure 5:**
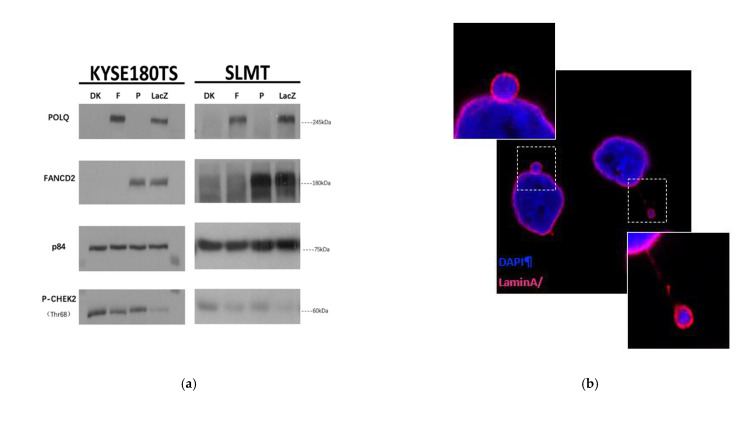

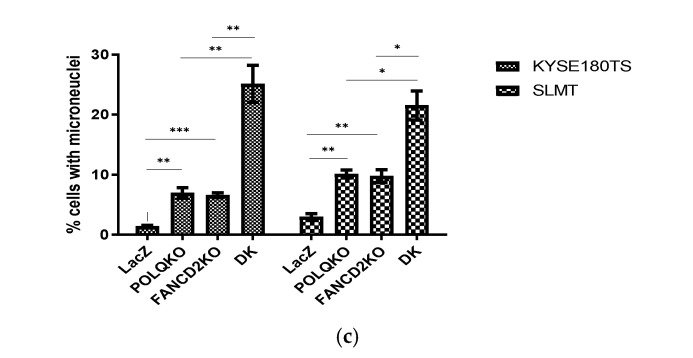
Double KO *POLQ* and *FANCD2* significantly induced genome instability and the formation of micronuclei. (**a**) Depleting *POLQ* and/or *FANCD2* exacerbated the level of CHEK2 phosphorylation. DK, *POLQ/FANCD2* double KO; F, *FANCD2* KO; P, *POLQ* KO; LacZ, negative control of CRISPR KO; p84, loading control. (**b**) A representative image of micronuclei. (**c**) Quantification of the percentage of cells with micronuclei in KYSE180TS and SLMT cell lines. Magnification: 63×/100× (zoomed-in image). Around 200 cells were counted for each group. Three independent experiments were performed. LacZ, negative control of CRISPR KO; DK, *POLQ/FANCD2* double KO. Student’s *t*-test, * *p* < 0.05; ** *p* < 0.01; *** *p* < 0.001. Data are presented as the mean ± SD.

To evaluate the activation status of CHEK2 in *POLQ*/*FANCD2* double KO cells, Western blotting was performed using KYSE180TS and SLMT cells. Figure 5a shows that CHEK2 was hyperphosphorylated upon single depletion of POLQ or FANCD2 in both cell lines. Double KO of these two genes caused an even higher level of phosphorylated CHEK2 when compared with either of the single KOs.

Micronuclei are often formed upon mis-segregation of DNA during cell division and are frequently associated with genomic instability [38,41]. Figure 5b shows representative micronuclei images. As shown in Figure 5c, the micronuclei were observed in only 1.4% of the control KYSE180TS cells, while 6.9% of POLQKO cells, 6.6% of FANCD2KO cells and 25.1% of *POLQ*/*FANCD2* double KO cells exhibited micronuclei. Similar results were found in the SLMT cell line as well. Micronuclei were observed in only 2.9% of the control SLMT cells, while 10.1% of POLQKO cells, 9.7% of FANCD2KO cells and 21.5% of *POLQ/FANCD2* double KO cells exhibited micronuclei (Figure 5c). These results imply that the loss of two DNA damage repair players, POLQ and FANCD2, leads to the substantial increase in genome instability and accumulation of cytosolic DNA.

### 3.6. Double Knockout POLQ and FANCD2 Induces the Expression of Interferon-Stimulated Genes (ISGs) and Upregulates cGAS and STAT1 Phosphorylation

**Figure 6:**
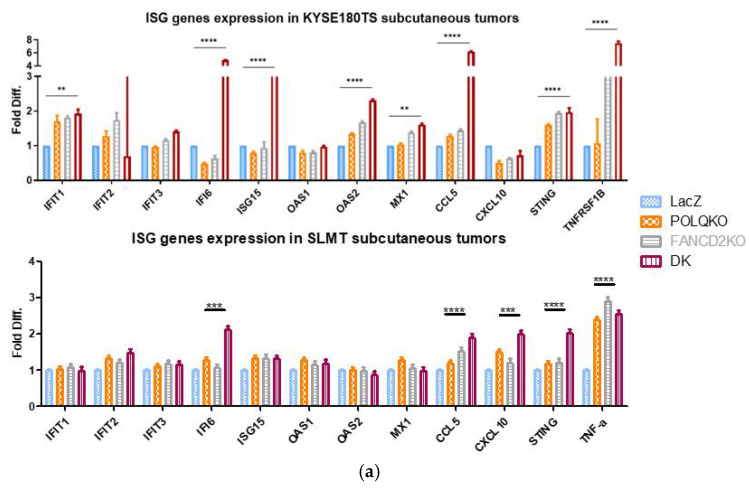

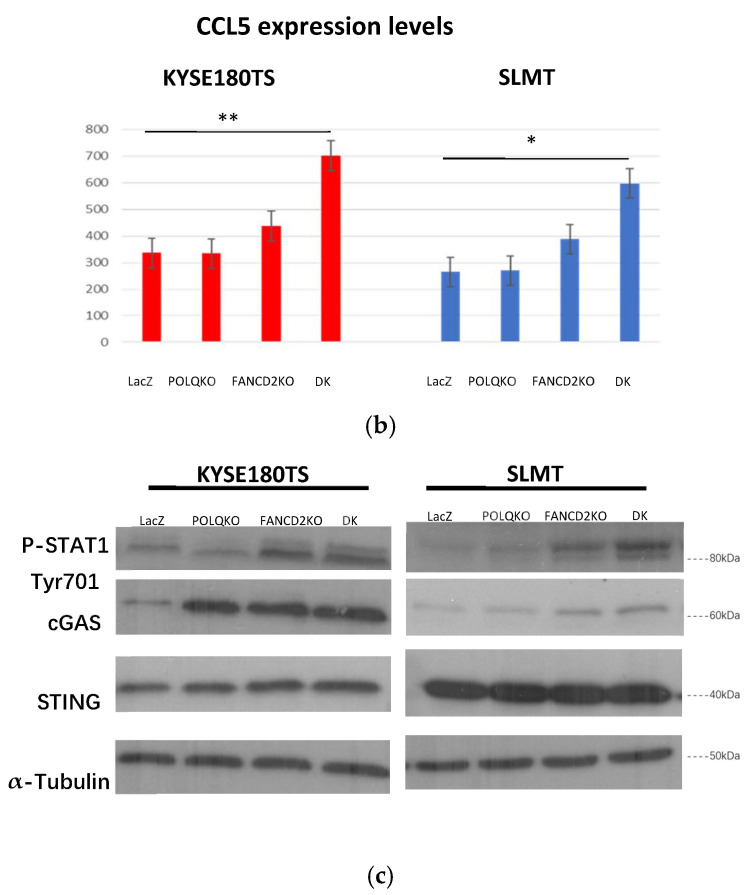
Double KO *POLQ* and *FANCD2* induced the expression of interferon-stimulated genes and upregulated cGAS and STAT1 phosphorylation. (**a**) ISGs mRNA expression levels in KYSE180TS and SLMT subcutaneous tumors. Student’s *t*-test, ** *p* < 0.01; *** *p* < 0.001; **** *p* < 0.0001. Data are presented as the mean ± SD. (**b**) CCL5 Protein level in KYSE180TS and SLMT cell lines. Student’s *t*-test, * *p* < 0.05; ** *p* < 0.01. (**c**) Activation of cGAS-STING-STAT1 pathway upon *POLQ/FANCD2* single/double KO. Data are presented as the mean ± SD.

The DNA damage repair deficiency has been recently linked to the activation of anti-tumor immunity by compelling evidence [14,15,16,17,18]. For example, the inactivation of *BRCA2* was reported to trigger the innate immune response [16]. We investigated the relationship between inactivating *POLQ* and/or *FANCD2*, two important DNA damage repair genes, and the potential activation of the innate immune response. ISGs play pivotal roles in enhancing innate immune responses [39]. A panel of ISGs was designed and their mRNA expression levels in subcutaneous mouse tumors inoculated with control, POLQKO, FANCD2KO and *POLQ*/*FANCD2* double KO ESCC cells were measured by qPCR. Compared with the control group, *POLQ/FANCD2* double KO subcutaneous tumors inoculated by KYSE180TS cell line had significantly higher levels of *IFIT1*, *IFI6*, *ISG15*, *OAS2*, *MX1*, *CCL5*, *STING* and *TNRSF1B* (Figure 6a). Similarly, ISGs (*IFI6, CCL5, CXCL10, STING* and *TNF-α*) were found to be upregulated in *POLQ/FANCD2* double KO SLMT subcutaneous tumors as compared with the controls (Figure 6a). No statistically significant difference in ISG expression levels was found between the single KO groups and the control group. The CCL5 was upregulated at the protein level in KYSE180TS and SLMT cells upon *POLQ*/*FANCD2* double KO (Figure 6b). CCL5 is a commonly used marker for activation of cytokine signaling [42]. Figure 6c shows the elevated phosphorylation of STAT1 and the hyperactivation of cGAS upon *POLQ/FANCD2* double KO. Taken together, these results indicate the potential activation of the innate immune response through the cGAS-STING-STAT1 pathway, after the loss of both POLQ and FANCD2 proteins.

## 4. Discussion

Encoded by *POLQ*, DNA polymerase theta (POLQ) has long been portrayed as a key player in mediating alternative end-joining repair of DNA DSBs [5]. The upregulation of *POLQ* has been observed in a variety of malignancies, including those of breast, lung, stomach, ovary and head and neck, and was associated with poor prognosis [8,9,10,12,43,44]. In this study, we discovered that *POLQ* is predominantly overexpressed in ESCC patient tumors at the mRNA level. In addition, after stratifying a cohort of 25 Hong Kong patients by the cause of death, a statistically significant negative correlation was uncovered between the relative *POLQ* mRNA expression levels in ESCC tumors and the patient survival upon surgical resection before ESCC-related death, highlighting the upregulation of *POLQ* association in ESCC tumors with growth advantages. In the in vivo tumorigenicity assay using KYSE180TS and SLMT cell lines, significantly smaller subcutaneous tumors were consistently observed in the *POLQ* KO groups, when compared with their respective controls. In line with these results, we also discovered that *POLQ* KO ESCC cells were sensitized to multiple genotoxic agents, as assessed in the MTT assay. Collectively, the current study is the first functional analysis suggesting that the upregulated *POLQ* in ESCC is associated with malignant phenotypes associated with poor prognosis.

Higher levels of DNA damage were found in POLQ-depleted ESCC cells than in control cells, especially under externally induced stresses. POLQ depletion concordantly enhanced the phosphorylation of CHK2 with or without the presence of DNA damage-inducing agents or ionizing radiation. In fact, whether *POLQ* promotes or suppresses genome instability remains largely controversial. Some biochemical studies have demonstrated that the involvement of *POLQ* in DNA damage repair is frequently accompanied by template insertion or large deletion, as *POLQ* has rather low fidelity in replication [45,46,47]. Conflicting evidence has been reported in the studies based on mouse or human systems. In some studies, the depletion of POLQ leads to increased DNA DSB formation, destabilized replication fork and elevated sensitivity to certain genotoxic drugs, suggesting the role of *POLQ* as a protector of genomic stability [8,48,49,50,51,52]. In other studies, however, POLQ depletion results in a reduced level of UV-induced mutation and chromosomal translocation, while *POLQ* overexpression lowers the replication fork speed, impairs cell cycle progression and increases the expression of DNA damage markers [53,54]. Recently, it has been proposed that the overexpressed *POLQ* neutralizes the excessive genome instability in cancer cells, which frequently possess higher levels of replication stress [55,56]. The function of *POLQ* concerning the genomic instability has not yet been described in the context of ESCC. Given the fact that *POLQ* is overexpressed in ESCC and is associated with poor clinical outcome, we postulated that the upregulated expression of *POLQ* may allow ESCC cells to better tolerate the increasing replication stress caused by uncontrolled proliferation or anti-cancer drugs and, therefore, promote the cancer progression.

As a critical component of the DNA damage repair orchestra, *FANCD2* functions in both the Fanconi anemia pathway that repairs inter-strand DNA crosslinks and the HR pathway that repairs DNA DSB breaks [8,57]. More importantly, FANCD2 is critical in the choice of DNA repair pathway at the replication fork and has been reported to facilitate polymerase theta recruitment during alt-EJ at the DNA damage sites [40]. By presenting the results showing the drastic inhibition of ESCC cell growth (both in vitro and in vivo) upon double KO of *POLQ* and *FANCD2*, our study also identified the potential synthetic lethality relationship between *POLQ* and *FANCD2* in ESCC. Since both the *FANCD2*-involved HR pathway and *POLQ*-mediated Alt-EJ pathway function in repairing DNA DSBs, efforts have been made to investigate the potential synthetic lethality relationship between *FANCD2* and *POLQ*. It was observed in ovarian carcinoma that POLQ depletion hindered the survival of FANCD2-deficient A2780 cells exposed to PARP inhibitors, cisplatin, and mitomycin C [8]. Consistently, the co-knockdown of *FANCD2* and *POLQ* in two lung cancer cell lines resulted in hypersensitivity to cisplatin, as compared with the single knockdown of *FANCD2* or *POLQ* [58]. Exacerbated levels of chromosomal breakage, checkpoint activation, and γH2AX phosphorylation in response to mitomycin C were also found upon POLQ-depletion in FANCD2-deficient cells [8]. Collectively, these in vitro findings suggest that FANCD2-deficient cancer cells are hypersensitive to inhibition of *POLQ*-mediated repair. In the in vivo context, despite the fact that *Fancd2−/−* and *Polq−/−* mice are viable and exhibit only subtle malignant phenotypes [49], viable *Fancd2–/–Polq–/–* mice were very uncommon from mating and frequently died prematurely due to severe congenital malformations [59]. It has also been reported that double knockdown of *POLQ* and *FANCD2* decreased the tumor volumes of xenotransplants of a human ovarian cancer cell line [8]. Our recent study revealed that *FANCD2* is also overexpressed in ESCC tumors compared with the normal tissues and the *FANCD2* single KO hindered DNA double-strand repair and inhibited ESCC cell growth both in vitro and in vivo [37]. As POLQ depletion also impairs the repair of DNA damage induced by multiple agents, we hypothesize that the double KO of *POLQ* and *FANCD2* gravely impairs the repair efficiency of DNA DSBs and promotes the genomic instability in ESCC to suppress ESCC cell proliferation in a synthetic lethal pattern.

Compelling evidence supports this linkage between the DNA damage repair deficiency and the innate immune responses. It has been reported that replication stress/DNA damage induced by ionizing radiation, cytotoxic drugs or defects in DNA damage repair genes may induce whole chromosome mis-segregation during mitosis [60]. As a result, the chromosome structures wrapped by their own nuclear membrane, also known as the micronuclei, occur in the cytoplasm [61]. The micronuclei are widely recognized as the consequence, and therefore, the marker of unresolved genome instability [17]. Meanwhile, such micronuclei have been described as a potential source of immunostimulatory cytosolic DNA, which is then recognized by the cytosolic nucleic acid sensor (cGAS). cGAMP then activates STING, which triggers transcriptional activation of interferon regulatory factor 3 (IRF3). IRF3 mediates the expression and then the secretion of proinflammatory cytokines like Type 1 IFN. On the one hand, the binding of the IFNs to their respective receptors activates Janus kinase 1 (JAK1) [21]. Then the players of the signal transducer and activator of the transcription family (STAT) are phosphorylated and later on trigger the expression of multiple interferon-stimulated genes (ISGs) like *CCL5*, *CXCL10*, *OAS1*, etc. [62]. On the other hand, type 1 IFN also helps in activating T cells by facilitating tumor antigen presentation of the dendritic cells. Lastly, activated T cells infiltrate tumors in response to chemokines like CXCL10 and recognize the presented tumor antigens [63,64]. In the present study, we showed that the double KO of *POLQ* and *FANCD2*, two important components of the DNA damage repair/replication stress response genes, leads to (1) an exacerbated level of micronuclei-harboring ESCC cells, (2) overexpression of ISGs including *CCL5* in ESCC cell lines and subcutaneous tumors, (3) upregulation of cGAS, and (4) exacerbated phosphorylation of STAT1 at Tyr 701. These all indicate the potential anti-tumor activation through a cGAS-STING-STAT1-mediated ISGs-involved pathway upon the loss of both POLQ and FANCD2 in ESCC. Results also suggest that the single depletion of POLQ or FANCD2 may also trigger this pathway, but in a much less intensive manner. To the best of our knowledge, this is the first study that reports the possible activation of the innate immune response caused by loss/deficiency of DNA damage repair proteins in esophageal cancers. It also suggests the potential of targeting POLQ and/or FANCD2 in combination with immunotherapy in the future management of ESCC. However, noting the huge differences between the human and mouse systems [65], particular attention is required when trying to extrapolate the mouse data from this study to human trials. It has been discovered that STING may have an intrinsic species–specific role as a receptor for an anti-cancer drug [66]. Meanwhile, the differences in transcriptional/post-transcriptional kinetics and the regulation of immune components between mouse and human may pose another challenge in translating mouse findings into clinical applications [67]. Further investigations are certainly warranted to validate this very important novel evidence to gain a deeper understanding of these findings.

## 5. Conclusions

By integrated analysis of one in-house and four public RNA-seq databases, we found that *POLQ* is predominantly upregulated in ESCC tumors. This ectopic expression of *POLQ* was also observed in a cohort of Hong Kong ESCC patients, in whom the expression level of *POLQ* was negatively correlated with the patient survival before ESCC-related death. The CRISPR technique was implemented to knock out *POLQ* in ESCC cell lines with high endogenous *POLQ* expression levels. The POLQ-depleted ESCC cells were significantly sensitized to stress inducers like hydroxyurea or platinum-based drugs compared with control cells. Both rH2AX foci staining and the comet assay indicated a higher level of genomic instability in *POLQ* KO cells than in control cells, when exposed to ionizing radiation. Double KO of *POLQ* and *FANCD2*, a DNA damage repair gene functioning in both Fanconi anemia and homologous recombination DNA damage repair pathways, significantly sabotaged cell proliferation in vitro as well as in vivo, as compared with either of these single KOs. Cells with POLQ and/or FANCD2 depletion also had exacerbated levels of CHK2 phosphorylation. A significantly increased number of micronuclei was observed in *POLQ* and/or *FANCD2* KO ESCC cells. Loss of *POLQ* and *FANCD2* also resulted in the activation of cGAS and upregulation of several interferon-stimulated genes (ISGs).

**Figure 7:**
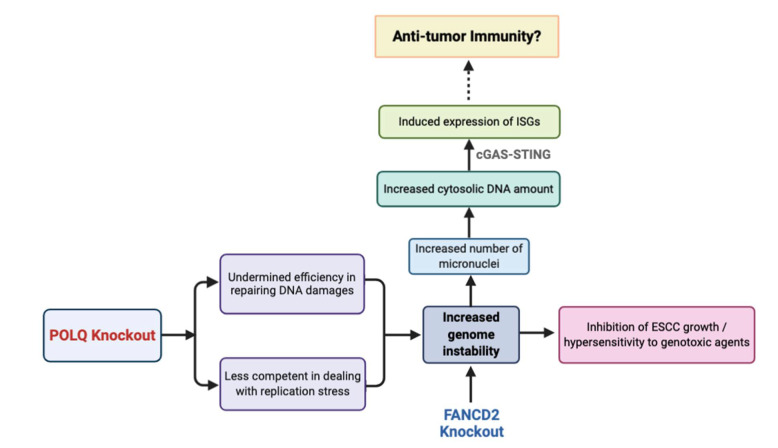
Schematic model for impact of *POLQ* and *FANCD2* KOs on genomic stability, genotoxin sensitivity, and anti-tumor immunity.

As summarized in Figure 7, the results of this study suggest the role of *POLQ* as a guardian of genome stability in ESCC. Meanwhile, the potential synthetic lethality relationship between *POLQ* and *FANCD2* in ESCC was described. More importantly, exciting novel findings from this study present new evidence linking the deficiencies of DNA damage repair genes (*POLQ*/*FANCD2*) with the activation of anti-tumor immunity through the cGAS-STING-STAT1 signaling pathway.

## Data Availability

The publicly available bulk RNA sequencing data for ESCC used in the study are available in the NCBI SRA database with the accession numbers SRP007169, SRP008496 and RP064894 (https://www.ncbi.nlm.nih.gov/sra/?term=SRP007169, https://www.ncbi.nlm.nih.gov/sra/?term=SRP008496, https://www.ncbi.nlm.nih.gov/sra/?term=SRP064894, accessed on 15 September 2015). The publicly available microarray data for ESCC were obtained in the NCBI GEO database with the accession number GSE23400 (https://www.ncbi.nlm.nih.gov/geo/query/acc.cgi?acc=GSE23400, accessed on 15 September 2015).

## Supplementary Materials

The following are available online at https://www.mdpi.com/article/10.3390/cancers13133204/s1, Figure S1: CRISPR KO of POLQ in representative ESCC cell lines, Figure S2: Basal levels of DNA damage in control and POLQ-depleted cells were measured without ionizing radiation treatment using rH2AX foci formation assay, Figure S3: Phosphorylation levels of major signaling checkpoints proteins in response to DNA damage upon POLQ KO measured by Western blotting in KYSE180TS cells, Figure S4: Validation of POLQ/FANCD2 double KO in ESCC cell lines by Western blotting, Figure S5–S14: Uncropped Western blot figures., Table S1: List of sgRNA oligos designed for functional KO of FANCD2 and POLQ using the CRISPR/Cas9 System, Table S2: List of the primers used for Q-PCR, Table S3: List of the antibodies used in the study.

## References

[1] Then E.O., Lopez M., Saleem S., Gayam V., Sunkara T., Culliford A., Gaduputi V. (2020). Esophageal Cancer: An Updated Surveillance Epidemiology and End Results Database Analysis. World J. Oncol..

[2] Abnet C.C., Arnold M., Wei W.-Q. (2018). Epidemiology of Esophageal Squamous Cell Carcinoma. Gastroenterol..

[3] Roshandel G., Nourouzi A., Pourshams A., Semnani S., Merat S., Khoshnia M. (2013). Endoscopic screening for esophageal squamous cell carcinoma. Arch. Iran. Med..

[4] Yousefzadeh M.J., Wood R.D. (2013). DNA polymerase POLQ and cellular defense against DNA damage. DNA Repair.

[5] Wood R.D., Doublié S. (2016). DNA polymerase θ (POLQ), double-strand break repair, and cancer. DNA Repair.

[6] Yoshimura M., Kohzaki M., Nakamura J., Asagoshi K., Sonoda E., Hou E., Prasad R., Wilson S.H., Tano K., Yasui A. (2006). Vertebrate POLQ and POLbeta cooperate in base excision repair of oxidative DNA damage. Mol. Cell..

[7] Koole W., Van Schendel R., Karambelas A.E., Van Heteren J.T., Okihara K.L., Tijsterman M. (2014). A Polymerase Theta-dependent repair pathway suppresses extensive genomic instability at endogenous G4 DNA sites. Nat. Commun..

[8] Ceccaldi R., Liu J.C., Amunugama R., Hajdu I., Primack B., Petalcorin M., O’Connor K., Konstantinopoulos P.A., Elledge S.J., Boulton S.J. (2015). Homologous-recombination-deficient tumours are dependent on Polθ-mediated repair. Nat. Cell Biol..

[9] Kawamura K., Bahar R., Seimiya M., Chiyo M., Wada A., Okada S., Hatano M., Tokuhisa T., Kimura H., Watanabe S. (2004). DNA polymerase θ is preferentially expressed in lymphoid tissues and upregulated in human cancers. Inter. J. Cancer.

[10] Pillaire M.-J., Selves J., Gordien K., Gouraud P.-A., Gentil C., Danjoux M., Do C., Negre V., Bieth A., Guimbaud R. (2010). A ‘DNA replication’signature of progression and negative outcome in colorectal cancer. Oncogene.

[11] Lessa R.C., Campos A.H., De Freitas C.E., Da Silva F.R., Kowalski L.P., Carvalho A., Vettore A.L. (2012). Identification of upregulated genes in oral squamous cell carcinomas. Head Neck.

[12] Lemée F., Bergoglio V., Fernandez-Vidal A., Machado-Silva A., Pillaire M.-J., Bieth A., Gentil C., Baker L., Martin A.-L., Leduc C. (2010). DNA polymerase θ up-regulation is associated with poor survival in breast cancer, perturbs DNA replication, and promotes genetic instability. Proc. Natl. Acad. Sci. USA.

[13] Ko J.M., Ning L., Zhao X., Chai A.W., Lei L.C., Choi S.S.A., Tao L., Law S., Kwong A., Lee N.P. (2020). BRCA2 loss-of-function germline mutations are associated with esophageal squamous cell carcinoma risk in Chinese. Int. J. Cancer.

[14] Parkes E.E., Walker S.M., Taggart L.E., McCabe N., Knight L.A., Wilkinson R., McCloskey K.D., Buckley N., Savage K.I., Salto-Tellez M. (2017). Activation of STING-Dependent Innate Immune Signaling By S-Phase-Specific DNA Damage in Breast Cancer. J. Natl. Cancer Inst..

[15] Pantelidou C., Sonzogni O., Taveira M.D.O., Mehta A.K., Kothari A., Wang D., Visal T., Li M.K., Pinto J., Castrillon J.A. (2019). PARP Inhibitor Efficacy Depends on CD8+ T-cell Recruitment via Intratumoral STING Pathway Activation in BRCA-Deficient Models of Triple-Negative Breast Cancer. Cancer Discov..

[16] Reisländer T., Lombardi E.P., Groelly F., Miar A., Porru M., Di Vito S., Wright B., Lockstone H., Biroccio A., Harris A. (2019). BRCA2 abrogation triggers innate immune responses potentiated by treatment with PARP inhibitors. Nat. Commun..

[17] MacKenzie K.J., Carroll P., Martin C.-A., Murina O., Fluteau A., Simpson D.J., Olova N., Sutcliffe H., Rainger J.K., Leitch A. (2017). cGAS surveillance of micronuclei links genome instability to innate immunity. Nat. Cell Biol..

[18] Erdal E., Haider S., Rehwinkel J., Harris A.L., McHugh P.J. (2017). A prosurvival DNA damage-induced cytoplasmic interferon response is mediated by end resection factors and is limited by Trex1. Genes Dev..

[19] Bose D. (2017). cGAS/STING Pathway in Cancer: Jekyll and Hyde Story of Cancer Immune Response. Int. J. Mol. Sci..

[20] Hellström K.E., Hellström I. (1969). Cellular Immunity Against Tumor Antigens. Adv. Cancer Res..

[21] Chen Q., Sun L., Chen Z.J. (2016). Regulation and function of the cGAS-STING pathway of cytosolic DNA sensing. Nat. Immunol..

[22] Wang H., Hu S., Chen X., Shi H., Chen C., Sun L., Chen Z.J. (2017). cGAS is essential for the antitumor effect of immune checkpoint blockade. Proc. Natl. Acad. Sci. USA.

[23] Leung A.C.C., Wong V.C.L., Yang L.C., Chan P.L., Daigo Y., Nakamura Y., Qi R.Z., Miller L.D., Liu E.T.-B., Wang L.D. (2008). Frequent decreased expression of candidate tumor suppressor gene, DEC1, and its anchorage-independent growth properties and impact on global gene expression in esophageal carcinoma. Int. J. Cancer.

[24] Kim D., Pertea G., Trapnell C., Pimentel H., Kelley R., Salzberg S.L. (2013). TopHat2: Accurate alignment of transcriptomes in the presence of insertions, deletions and gene fusions. Genome Biol..

[25] Yu Y., Cao J., Wu W., Zhu Q., Tang Y., Zhu C., Dai J., Li Z., Wang J., Xue L. (2019). Genome-wide copy number variation analysis identified ANO1 as a novel oncogene and prognostic biomarker in esophageal squamous cell cancer. Carcinog..

[26] Young L., Sung J., Stacey G., Masters J.R. (2010). Detection of Mycoplasma in cell cultures. Nat. Protoc..

[27] Yu V.Z., Ko J.M.Y., Ning L., Dai W., Law S., Lung M.L. (2019). Endoplasmic reticulum-localized ECM1b suppresses tumor growth and regulates MYC and MTORC1 through modulating MTORC2 activation in esophageal squamous cell carcinoma. Cancer Lett..

[28] Yu V.Z., Wong V.C.-L., Dai W., Ko J.M.-Y., Lam A.K.-Y., Chan K.W., Samant R.S., Lung H.L., Shuen W.H., Law S. (2015). Nuclear Localization of DNAJB6 Is Associated With Survival of Patients With Esophageal Cancer and Reduces AKT Signaling and Proliferation of Cancer Cells. Gastroenterol..

[29] Kearns N.A., Genga R.M.J., Enuameh M.S., Garber M., Wolfe S.A., Maehr R. (2014). Cas9 effector-mediated regulation of transcription and differentiation in human pluripotent stem cells. Development.

[30] Lo P.H.Y., Ko J.M.Y., Yu Z.Y., Law S., Wang L.D., Li J.-L., Srivastava G., Tsao S.W., Stanbridge E.J., Lung M.L. (2012). The LIM domain protein, CRIP2, promotes apoptosis in esophageal squamous cell carcinoma. Cancer Lett..

[31] Lung H.L., Bangarusamy D.K., Xie D., Cheung A.K.L., Cheng Y., Kumaran M.K., Miller L., Liu E.T.-B., Guan X.-Y., Sham J.S. (2005). THY1 is a candidate tumour suppressor gene with decreased expression in metastatic nasopharyngeal carcinoma. Oncogene.

[32] Fernandez-Vidal A., Guitton-Sert L., Cadoret J.-C., Drac M., Schwob E., Baldacci G., Cazaux C., Hoffmann J.-S. (2014). A role for DNA polymerase θ in the timing of DNA replication. Nat. Commun..

[33] Olive P.L., Banáth J.P. (2006). The comet assay: A method to measure DNA damage in individual cells. Nat. Protoc..

[34] Gyori B.M., Venkatachalam G., Thiagarajan P., Hsu D., Clement M.-V. (2014). OpenComet: An automated tool for comet assay image analysis. Redox Biol..

[35] Langie S.A.S., Azqueta A., Collins A.R. (2015). The comet assay: Past, present, and future. Front. Genet..

[36] Zhou C., Li Z., Diao H., Yu Y., Zhu W., Dai Y., Chen F.F., Yang J. (2006). DNA damage evaluated by gammaH2AX foci formation by a selective group of chemical/physical stressors. Mutation Res..

[37] Lei L.C., Yu V.Z., Ko J.M.Y., Ning L., Lung M.L. (2020). FANCD2 Confers a Malignant Phenotype in Esophageal Squamous Cell Carcinoma by Regulating Cell Cycle Progression. Cancers.

[38] Michl J., Zimmer J., Buffa F., McDermott U., Tarsounas M. (2016). FANCD2 limits replication stress and genome instability in cells lacking BRCA2. Nat. Struct. Mol. Biol..

[39] Schneider W.M., Chevillotte M.D., Rice C.M. (2014). Interferon-Stimulated Genes: A Complex Web of Host Defenses. Annu. Rev. Immunol..

[40] Kais Z., Rondinelli B., Holmes A., O’Leary C., Kozono D., D’Andrea A.D., Ceccaldi R. (2016). FANCD2 Maintains Fork Stability in BRCA1/2-Deficient Tumors and Promotes Alternative End-Joining DNA Repair. Cell Rep..

[41] Kee Y., D’Andrea A.D. (2010). Expanded roles of the Fanconi anemia pathway in preserving genomic stability. Genes Dev..

[42] Harding S., Benci J., Irianto J., Discher D.E., Minn A.J., Greenberg R.A. (2017). Mitotic progression following DNA damage enables pattern recognition within micronuclei. Nat. Cell Biol..

[43] Leoncini E., Ricciardi W., Cadoni G., Arzani D., Petrelli L., Paludetti G., Brennan P., Luce D., Stucker I., Matsuo K. (2013). Adult height and head and neck cancer: A pooled analysis within the INHANCE Consortium. Eur. J. Epidemiol..

[44] Allera-Moreau C., Rouquette I., Lepage B., Oumouhou N., Walschaerts M., Leconte E., Schilling V., Gordien K., Brouchet L., Delisle M.B. (2012). DNA replication stress response involving PLK1, CDC6, POLQ, RAD51 and CLASPIN upregulation prognoses the outcome of early/mid-stage non-small cell lung cancer patients. Oncogenesis.

[45] Sallmyr A., Tomkinson A.E. (2018). Repair of DNA double-strand breaks by mammalian alternative end-joining pathways. J. Biol. Chem..

[46] Schimmel J., van Schendel R., Dunnen J.T.D., Tijsterman M. (2019). Templated Insertions: A Smoking Gun for Polymerase Theta-Mediated End Joining. Trends Genet..

[47] Seki M., Masutani C., Yang L.W., Schuffert A., Iwai S., Bahar I., Wood R.D. (2004). High-efficiency bypass of DNA damage by human DNA polymerase Q. EMBO J..

[48] Goff J.P., Shields D.S., Seki M., Choi S., Epperly M.W., Dixon T., Wang H., Bakkenist C.J., Dertinger S.D., Torous D.K. (2009). Lack of DNA polymerase θ (POLQ) radiosensitizes bone marrow stromal cells in vitro and increases retic-ulocyte micronuclei after total-body irradiation. Radiat. Res..

[49] Shima N., Munroe R.J., Schimenti J.C. (2004). The Mouse Genomic Instability Mutation chaos1 Is an Allele of Polq That Exhibits Genetic Interaction with Atm. Mol. Cell. Biol..

[50] Kelso A.A., Lopezcolorado F.W., Bhargava R., Stark J.M. (2019). Distinct roles of RAD52 and POLQ in chromosomal break repair and replication stress response. PLoS Genet..

[51] Yousefzadeh M.J., Wyatt D., Takata K.-I., Mu Y., Hensley S.C., Tomida J., Bylund G.O., Doublie S., Johansson E., Ramsden D.A. (2014). Mechanism of Suppression of Chromosomal Instability by DNA Polymerase POLQ. PLoS Genet..

[52] Higgins G.S., Prevo R., Lee Y.-F., Helleday T., Muschel R.J., Taylor T., Yoshimura M., Hickson I.D., Bernhard E.J., McKenna W.J. (2010). A small interfering RNA screen of genes involved in DNA repair identifies tumor-specific radiosensi-tization by POLQ knockdown. Cancer Res..

[53] Mateos-Gomez P.A., Gong F., Nair N., Miller K.M., Denchi E.L., Sfeir A. (2015). Mammalian polymerase θ promotes alternative NHEJ and suppresses recombination. Nat. Cell Biol..

[54] Yoon J.-H., McArthur M.J., Park J., Basu D., Wakamiya M., Prakash L., Prakash S. (2019). Error-Prone Replication through UV Lesions by DNA Polymerase θ Protects against Skin Cancers. Cell.

[55] Franchet C., Hoffmann J.-S. (2019). When RAD52 Allows Mitosis to Accept Unscheduled DNA Synthesis. Cancers.

[56] Maiorano D., El Etri J., Franchet C., Hoffmann J.-S. (2021). Translesion Synthesis or Repair by Specialized DNA Polymerases Limits Excessive Genomic Instability upon Replication Stress. Int. J. Mol. Sci..

[57] Moldovan G.-L., D’Andrea A.D. (2009). How the Fanconi Anemia Pathway Guards the Genome. Annu. Rev. Genet..

[58] Dai C.-H., Chen P., Li J., Lan T., Chen Y.-C., Qian H., Chen K., Li M.-Y. (2016). Co-inhibition of pol θ and HR genes efficiently synergize with cisplatin to suppress cisplatin-resistant lung cancer cells survival. Oncotarget.

[59] Parmar K., Kim J., Sykes S.M., Shimamura A., Stuckert P., Zhu K., Hamilton A., Deloach M.K., Kutok J.L., Akashi K. (2010). Hematopoietic Stem Cell Defects in Mice with Deficiency of Fancd2 or Usp1. STEM CELLS.

[60] Wilhelm T., Olziersky A.-M., Harry D., De Sousa F., Vassal H., Eskat A., Meraldi P. (2019). Mild replication stress causes chromosome mis-segregation via premature centriole disengagement. Nat. Commun..

[61] He B., Gnawali N., Hinman A.W., Mattingly A.J., Osimani A., Cimini D. (2019). Chromosomes missegregated into micronuclei contribute to chromosomal instability by missegregating at the next division. Oncotarget.

[62] Schoggins J.W., Wilson S.J., Panis M., Murphy M.Y., Jones C.T., Bieniasz P., Rice C.M. (2011). A diverse range of gene products are effectors of the type I interferon antiviral response. Nat. Cell Biol..

[63] MacMicking J.D. (2012). Interferon-inducible effector mechanisms in cell-autonomous immunity. Nat. Rev. Immunol..

[64] Zitvogel L., Galluzzi L., Kepp O., Smyth M., Kroemer G. (2015). Type I interferons in anticancer immunity. Nat. Rev. Immunol..

[65] Zschaler J., Schlorke D., Arnhold J. (2014). Differences in innate immune response between man and mouse. Crit. Rev. Immunol..

[66] Conlon J., Burdette D.L., Sharma S., Bhat N., Thompson M., Jiang Z., Rathinam V.A.K., Monks B., Jin T., Xiao T.S. (2013). Mouse, but not Human STING, Binds and Signals in Response to the Vascular Disrupting Agent 5,6-Dimethylxanthenone-4-Acetic Acid. J. Immunol..

[67] Bose D., Neumann A., Timmermann B., Meinke S., Heyd F. (2019). Differential Interleukin-2 Transcription Kinetics Render Mouse but Not Human T Cells Vulnerable to Splicing Inhibition Early after Activation. Mol. Cell. Biol..

