# Supplementary material for: Depletion of DNA Polymerase Theta Inhibits Tumor Growth and Promotes Genome Instability through the cGAS-STING-ISG Pathway in Esophageal Squamous Cell Carcinoma
Source: Cancers (Basel). 2021 Jun 26;13(13):3204. doi: 10.3390/cancers13133204 (PMC8268317; doi:10.3390/cancers13133204)
Supplement: Supplementary file 1 [file cancers-13-03204-s001.zip › cancers-1228178-supplementary.pdf]

cancers

MDPI

Supplementary Materials: Depletion of DNA Polymerase Theta Inhibits Tumor Growth and Promotes Genome Instability through the cGAS-STING-ISG Pathway in Esophageal Squamous Cell Carcinoma

Jian Li, Josephine Mun-Yee Ko, Wei Dai, Valen Zhuoyou Yu, Hoi Yan Ng, Jean-Sébastien Hoffmann and Maria Li Lung

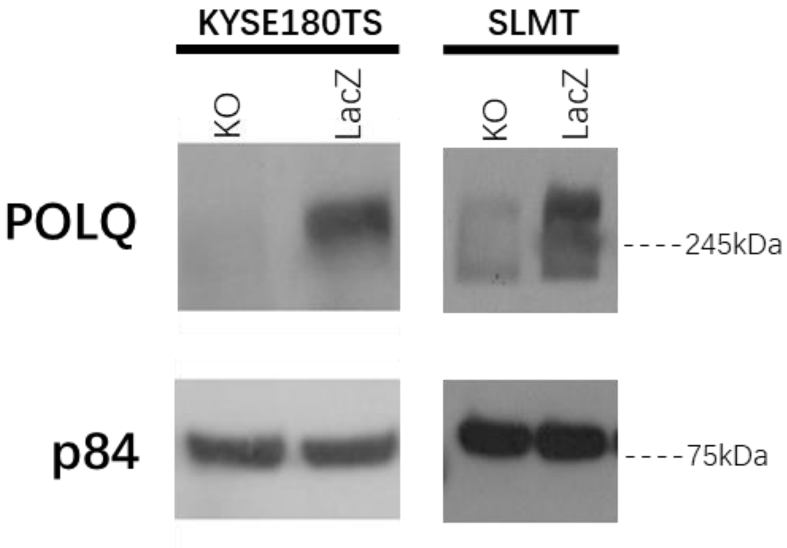

Figure S1. CRISPR KO of *POLQ* in representative ESCC cell lines. LacZ, negative control of CRISPR KO; p84 loading control.

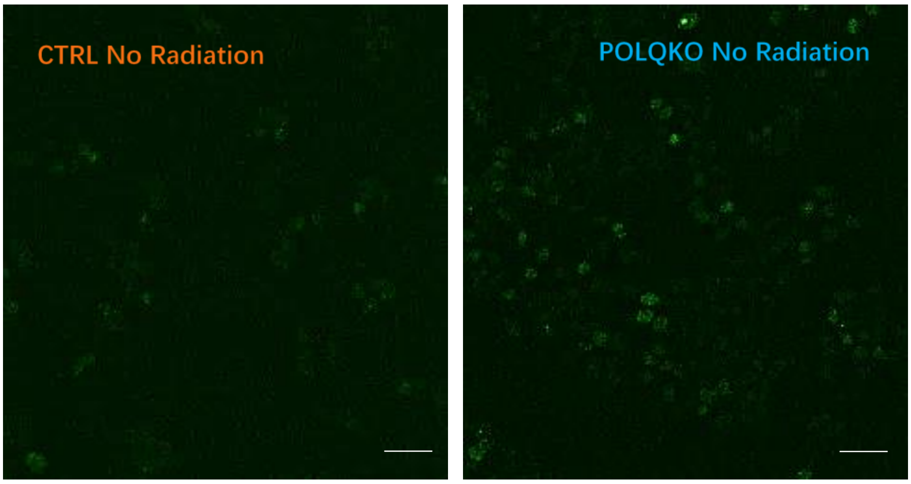

Figure S2. Basal levels of DNA damage in control and POLQ-depleted cells were measured without ionizing radiation treatment using rH2AX foci formation assay. Scale bar, 50 μm.

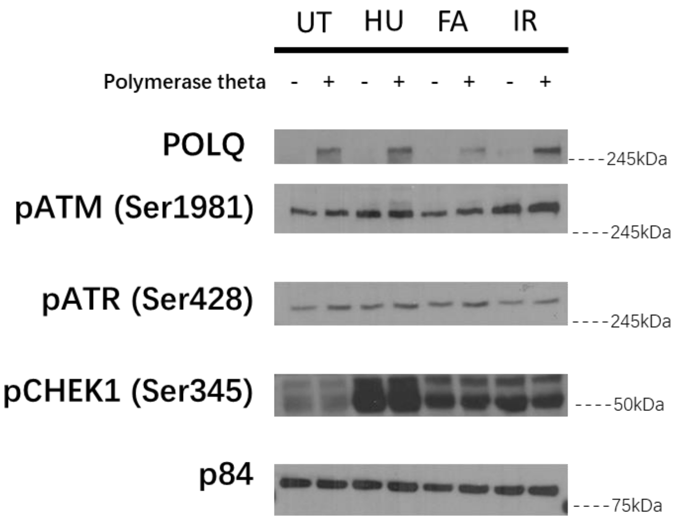

Figure S3. The phosphorylation levels of major signaling checkpoints proteins in response to DNA damage upon *POLQ* KO measured by western blotting in KYSE180TS cells. UT, untreated; FA, 1mM formaldehyde; HU, 4mM hydroxyurea; IR, 4Gy ionizing radiation; LacZ, negative control of CRISPR KO; p84, loading control.

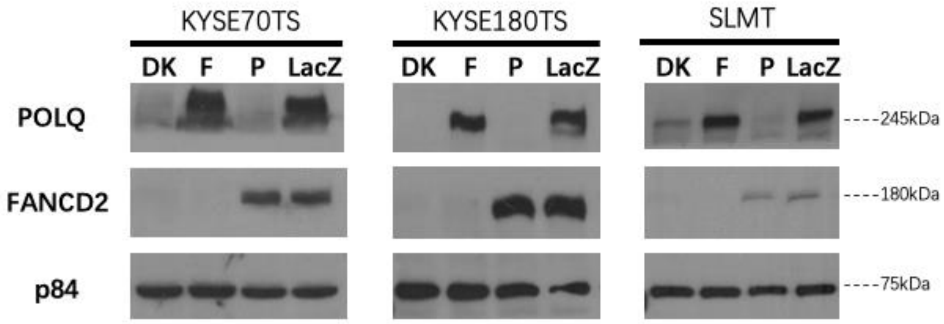

Figure S4. Validation of *POLQ*/*FANCD2* double KO in ESCC cell lines by western blotting. DK, *POLQ*/*FANCD2* double KO; F, *FANCD2* KO; P, *POLQ* KO; LacZ, negative control of CRISPR KO; p84, loading control.

(a)

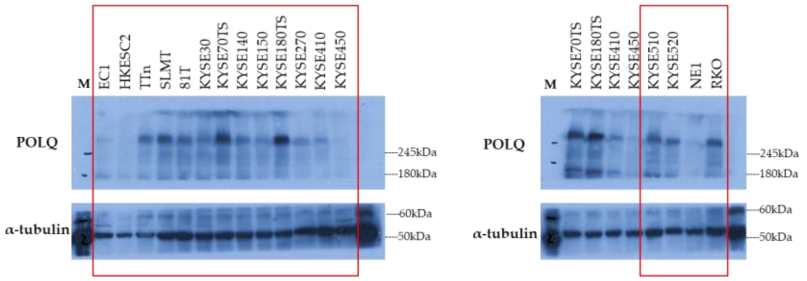

(b)

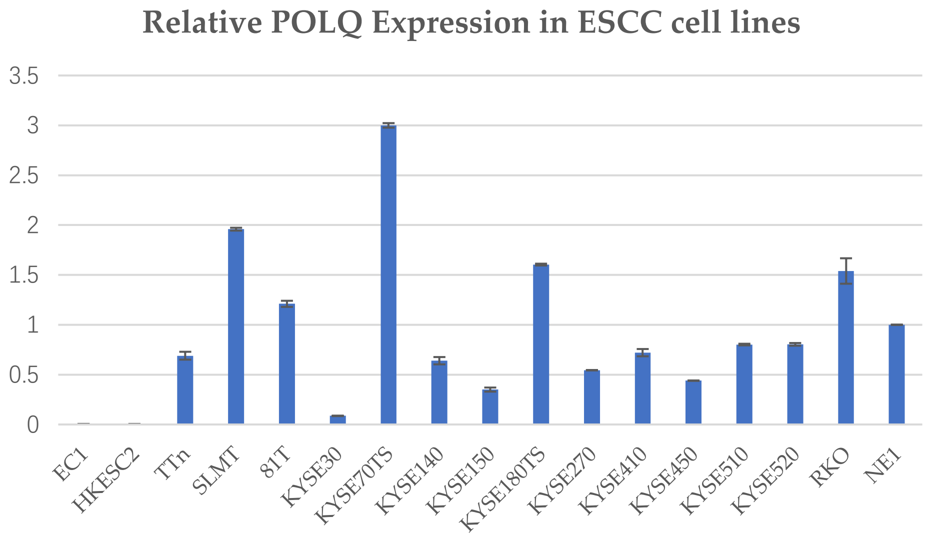

Figure S5. The original Western blots and band quantifications corresponding to Figure 1f. (**a**) Whole length western blots shown in Figure 1f. Red box encircles the bands shown in the main text. (**b**) Intensity ratio of each band normalized with loading control α-tubulin and the control cell line NE1 (n=2, data presented as the mean ± SD). M, molecular weight markers.

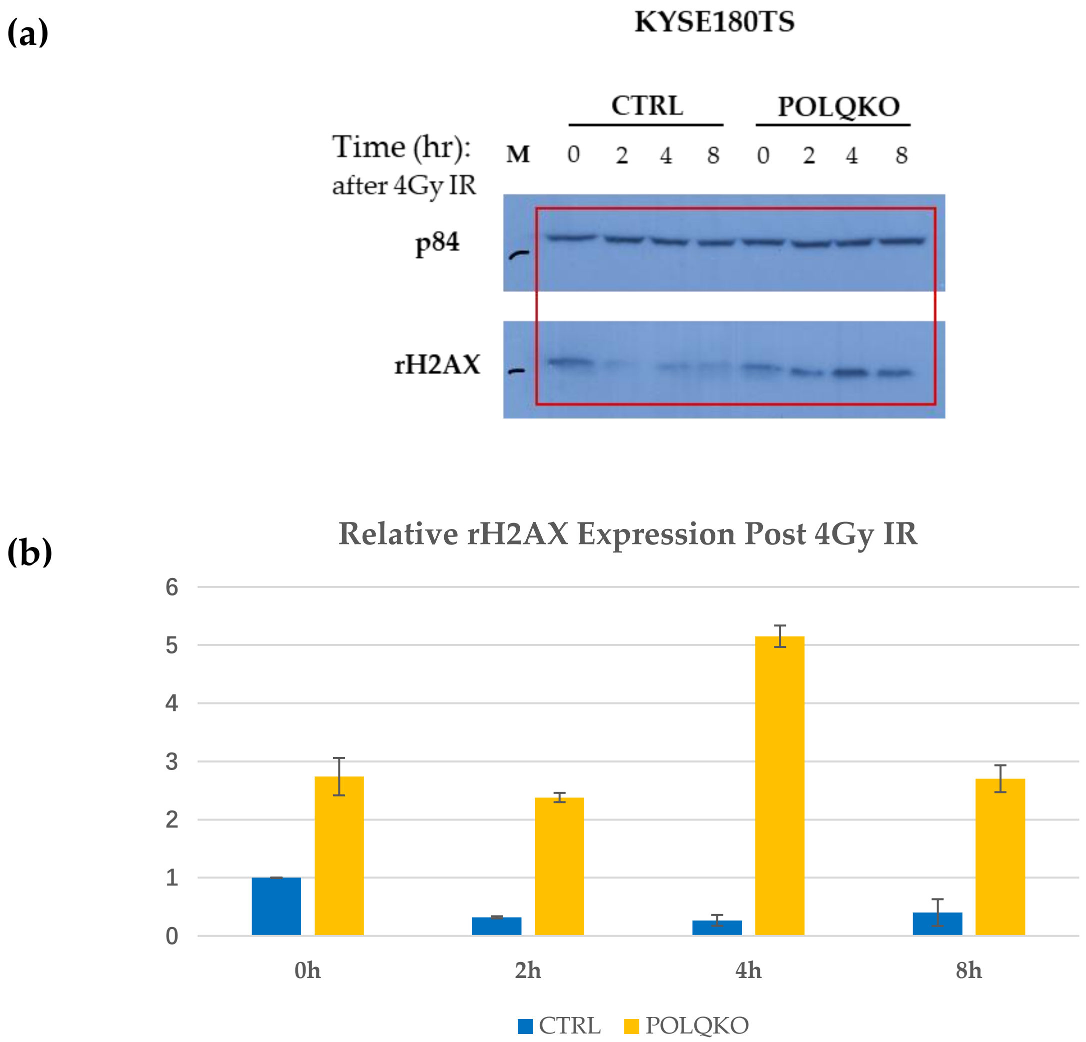

Figure S6. The original Western blots and band quantifications corresponding to Figure 2d. (**a**) Whole length western blots shown in Figure 2d. Red box encircles the bands shown in the main text. (**b**) Intensity ratio of each band normalized with loading control p84 and the control cell line (n=3, data presented as the mean ± SD). IR, ionizing radiation. M, molecular weight markers.

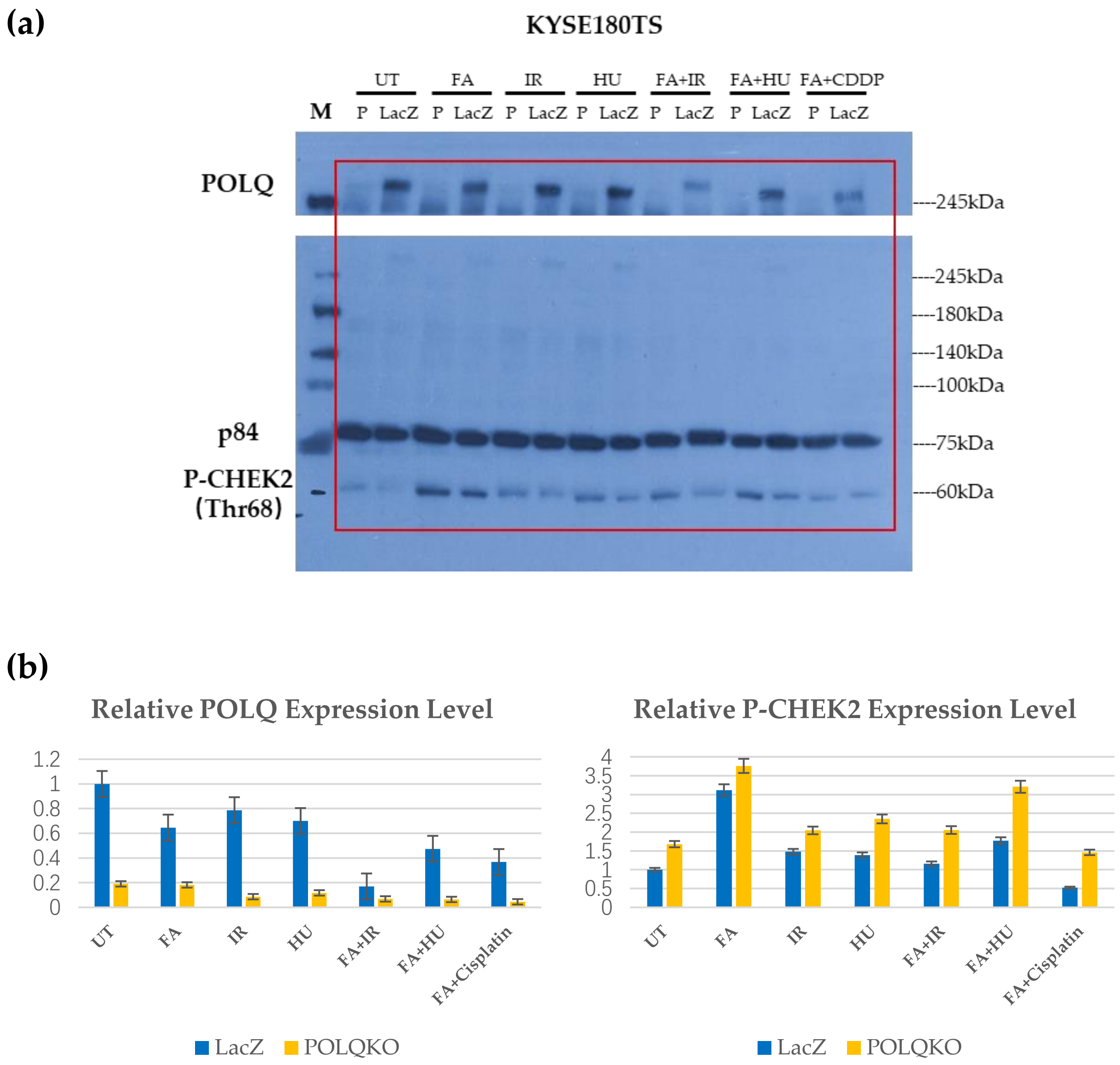

Figure S7. The original Western blots and band quantifications corresponding to Figure 2e. (**a**) Whole length western blots shown in Figure 2e. Red box encircles the bands shown in the main text. (**b**) Intensity ratio of each band normalized with loading control p84 and the untreated control cell line (n=2, data presented as the mean ± SD). M, molecular weight markers. P, *POLQ* KO. LacZ, negative control of CRISPR KO. UT, untreated; FA, 1mM formaldehyde; IR, 4Gy ionizing radiation; HU, 4mM hydroxyurea; CDDP, 2uM cisplatin.

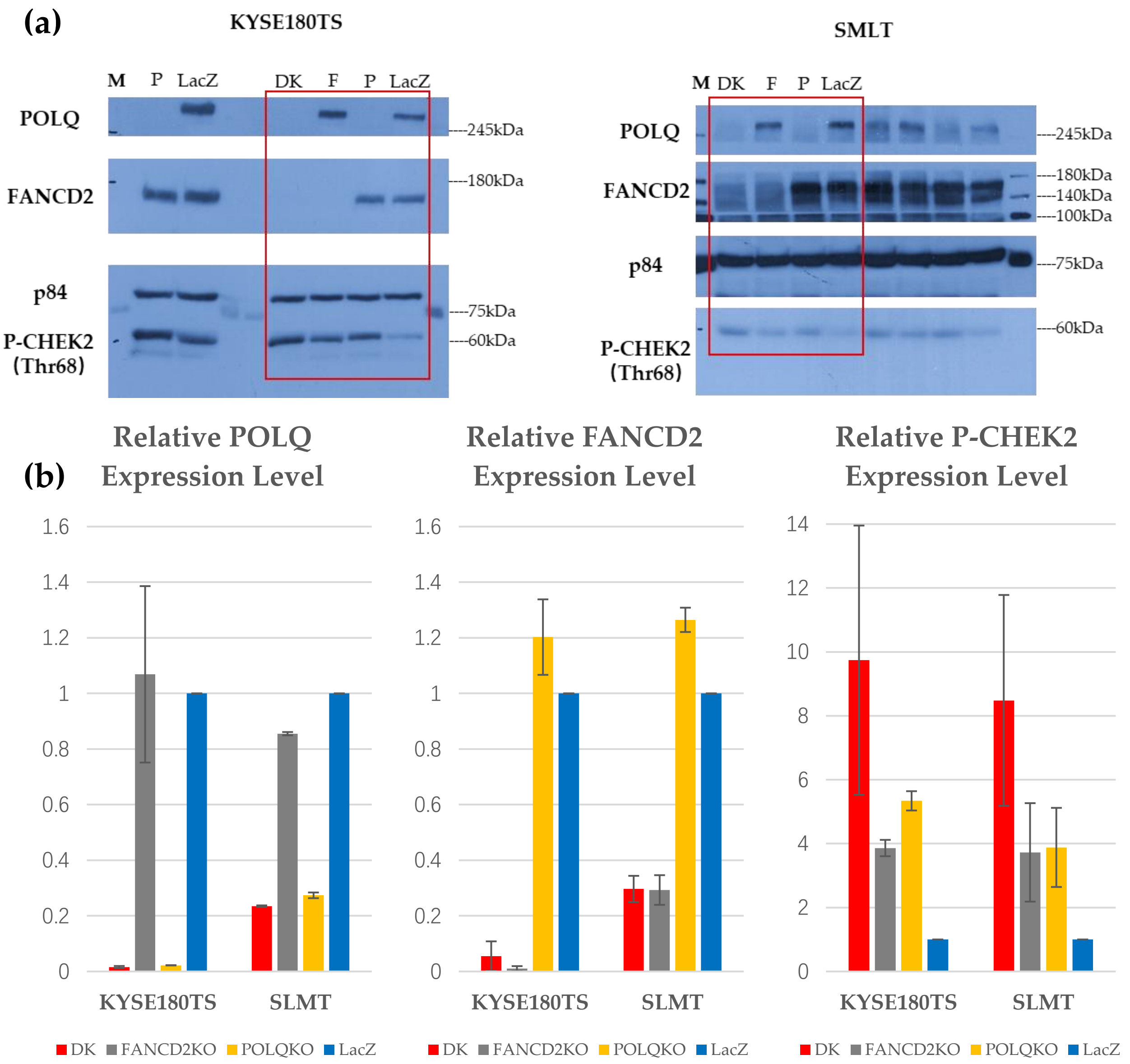

Figure S8. The original Western blots and band quantifications corresponding to Figure 5a. (**a**) Whole length western blots shown in Figure 5a. Red box encircles the bands shown in the main text. (**b**) Intensity ratio of each band normalized with loading control p84 and the LacZ control cell line (n=3, data presented as the mean ± SD). M, molecular weight markers; DK, *POLQ*/*FANCD2* double KO; F, *FANCD2* KO; P, *POLQ* KO; LacZ, negative control of CRISPR KO.

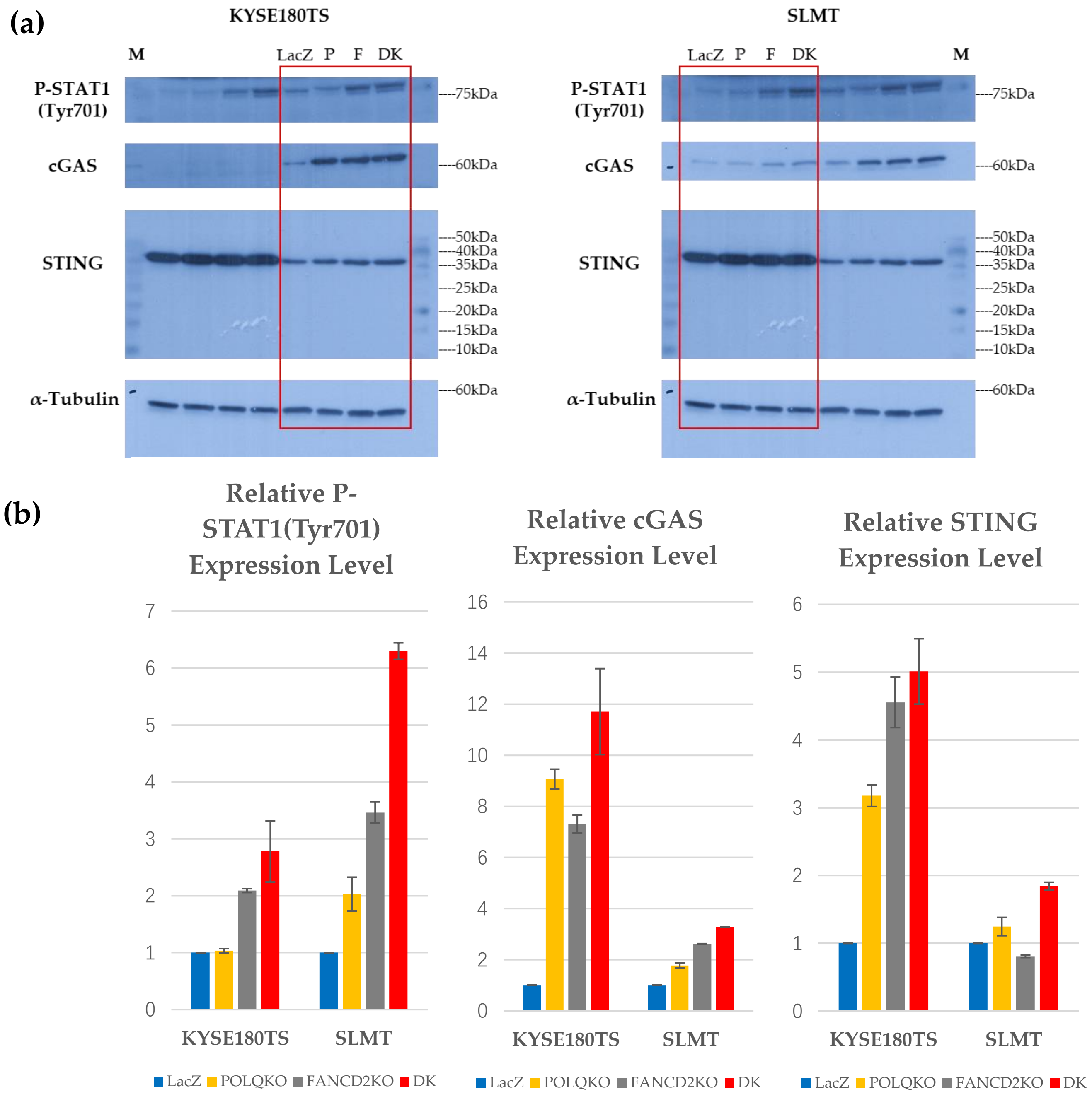

Figure S9. The original Western blots and band quantifications corresponding to Figure 6c. (**a**) Whole length western blots shown in Figure 6c. Red box encircles the bands shown in the main text. (**b**) Intensity ratio of each band normalized with loading control α-tubulin and the LacZ control cell line (n=2, data presented as the mean ± SD). M, molecular weight markers; DK, *POLQ*/*FANCD2* double KO; F, *FANCD2* KO; P, *POLQ* KO; LacZ, negative control of CRISPR KO.

(a)

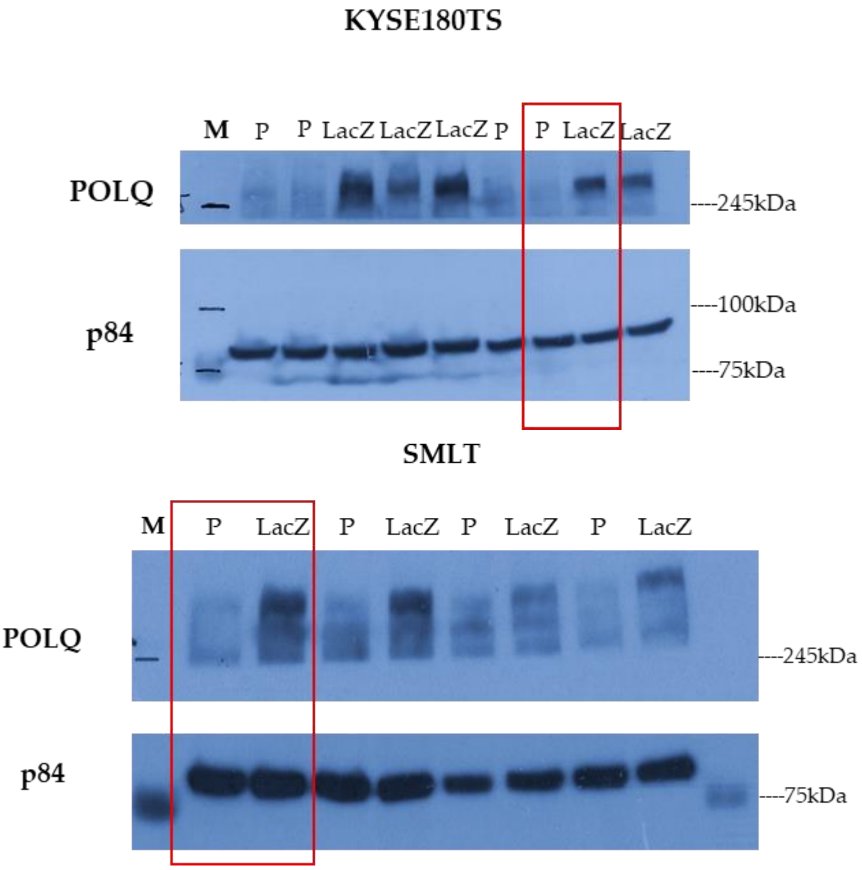

(b)

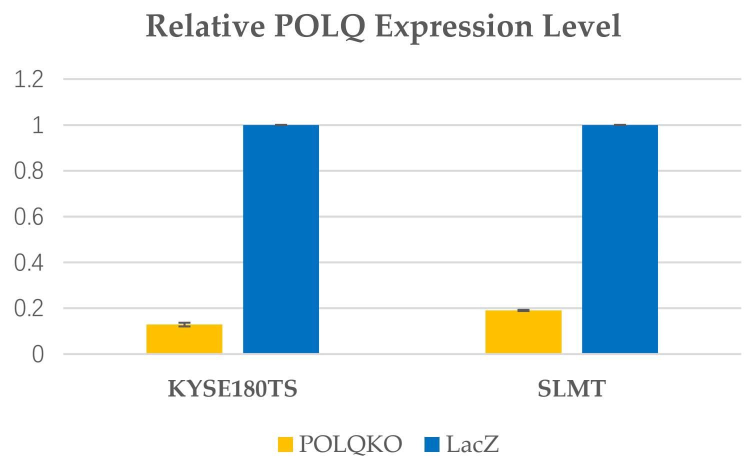

Figure S10. The original Western blots and band quantifications corresponding to Figure S1. (**a**) Whole length western blots shown in Figure S1. Red box encircles the bands shown in the supplementary materials. (**b**) Intensity ratio of each band normalized with loading control p84 and the LacZ control cell line (n=2, data presented as the mean ± SD). M, molecular weight markers; P, *POLQ* KO; LacZ, negative control of CRISPR KO.

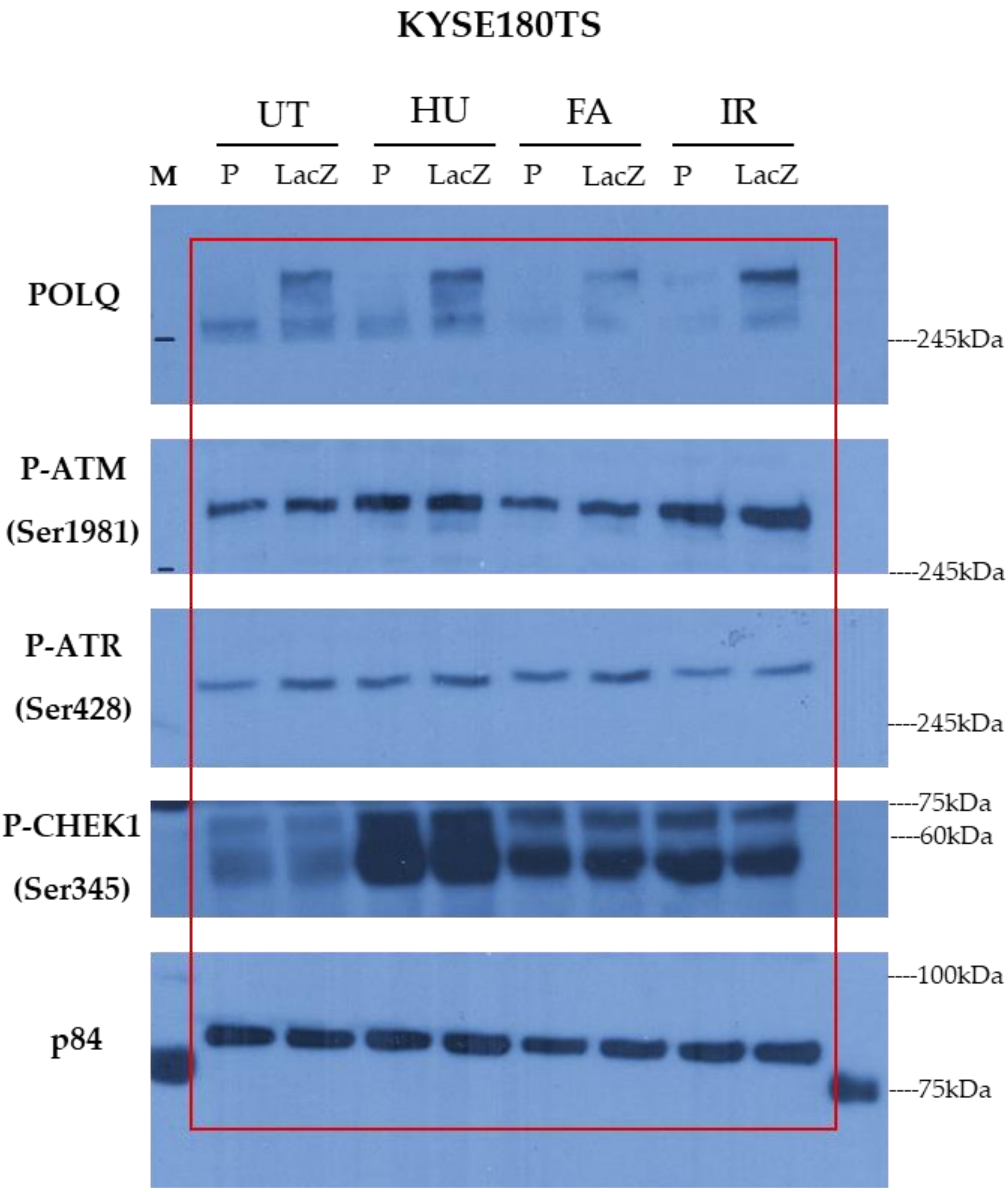

Figure S11. The original Western blots corresponding to Figure S3. Red box encircles the bands shown in the supplementary materials. M, molecular weight markers; P, *POLQ* KO; LacZ, negative control of CRISPR KO. UT, untreated; HU, 4mM hydroxyurea; FA, 1mM formaldehyde; IR, 4Gy ionizing radiation.

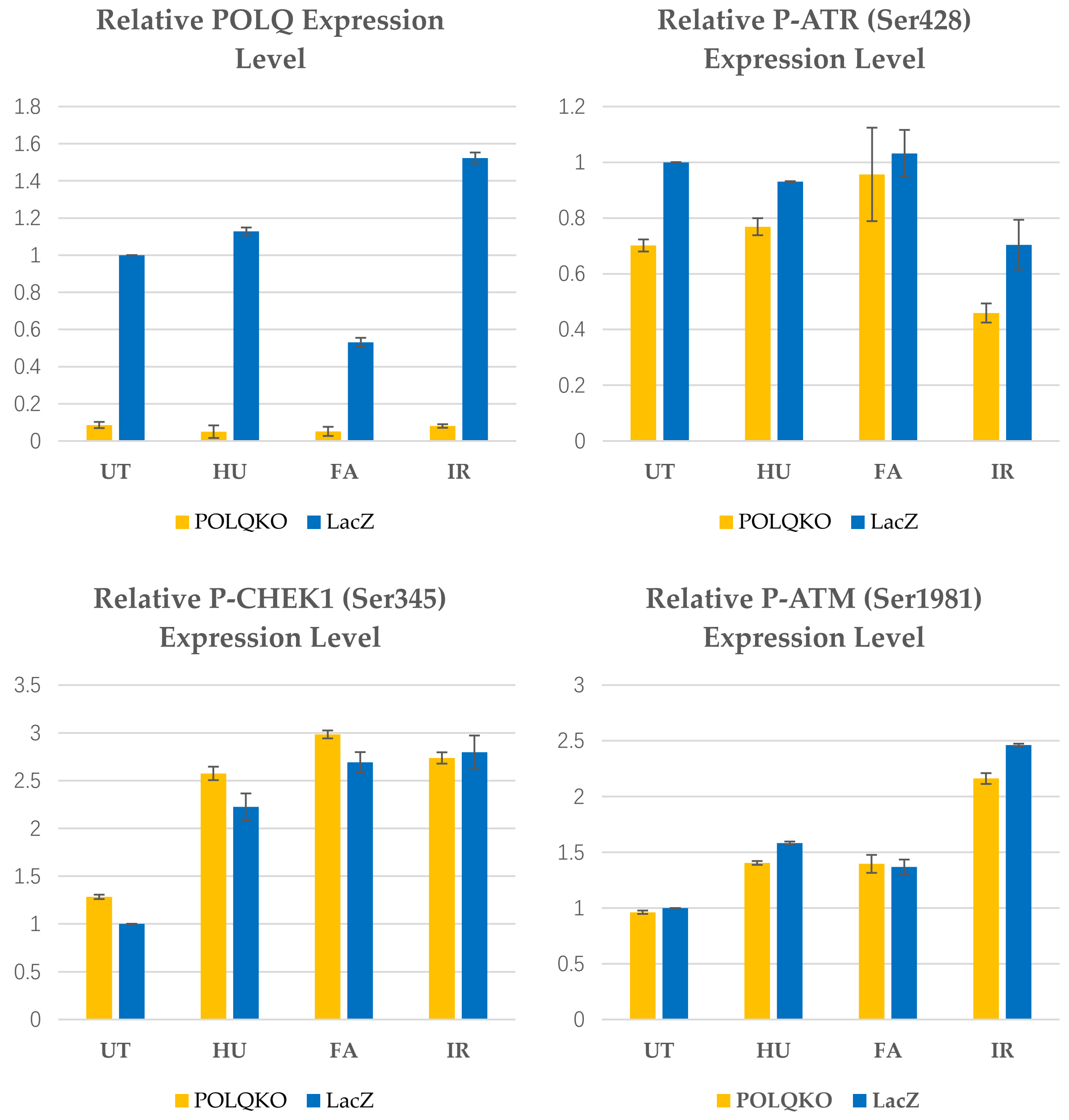

Figure S12. Band quantifications corresponding to Figure S3. Intensity ratio of each band normalized with loading control p84 and the LacZ control cell line (n=2, data presented as the mean ± SD). LacZ, negative control of CRISPR KO. UT, untreated; HU, 4mM hydroxyurea; FA, 1mM formaldehyde; IR, 4Gy ionizing radiation.

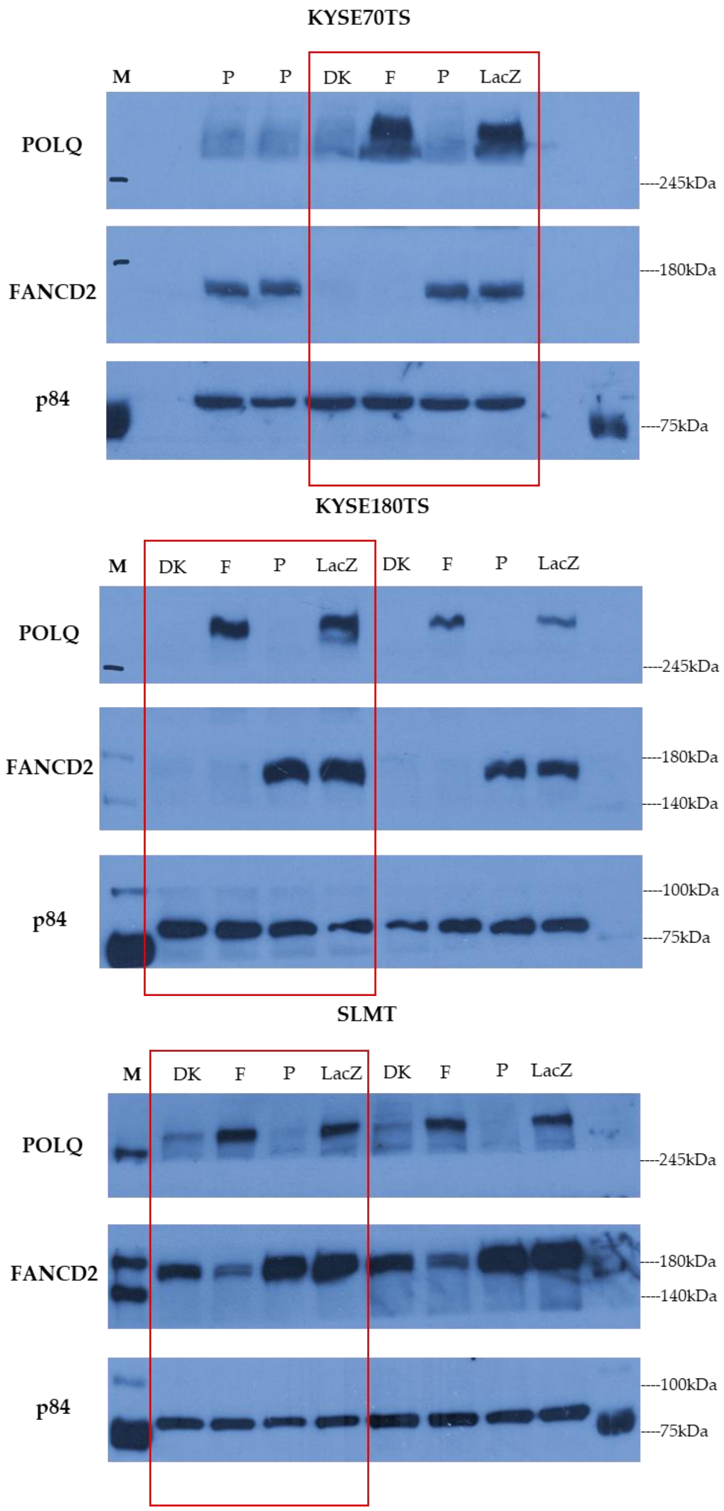

Figure S13. The original Western blots corresponding to Figure S4. Red box encircles the bands shown in the supplementary materials. M, molecular weight markers; DK, *POLQ*/*FANCD2* double KO; F, *FANCD2* KO; P, *POLQ* KO; LacZ, negative control of CRISPR KO.

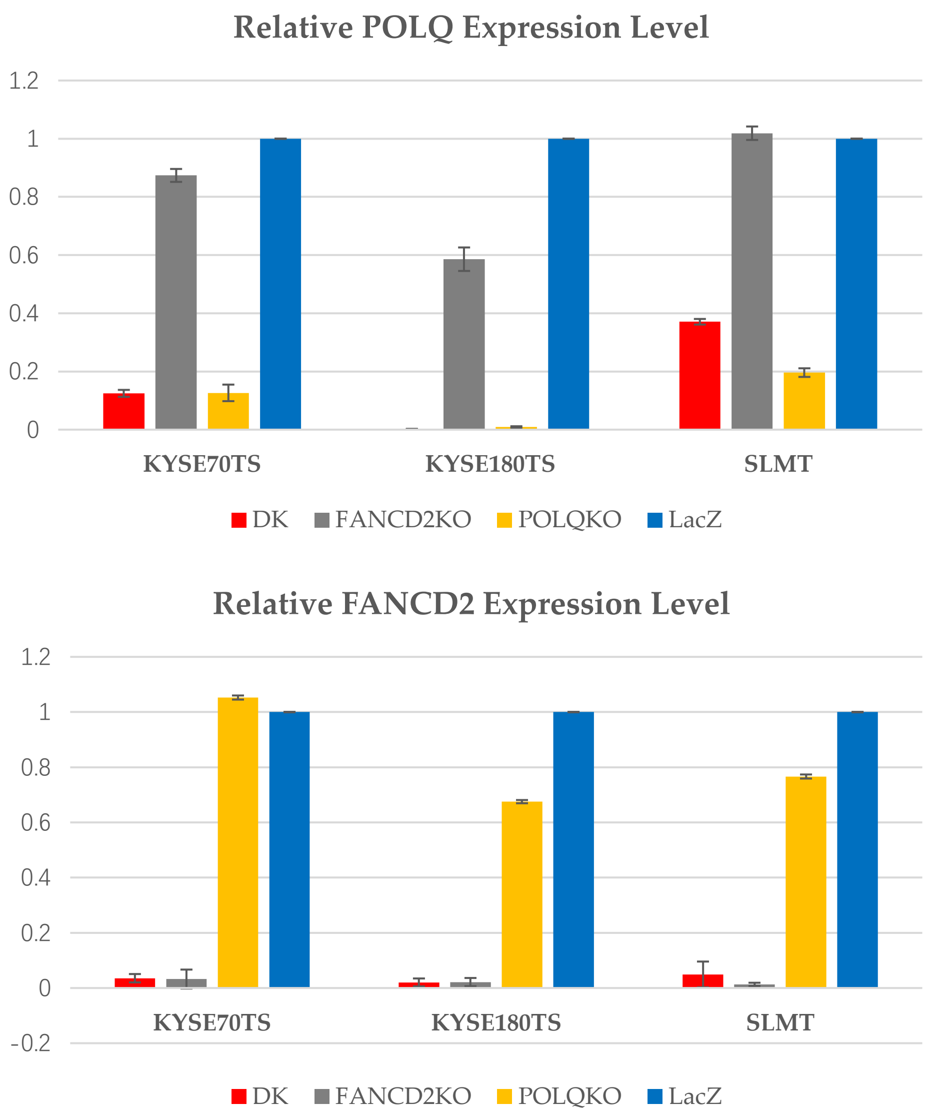

Figure S14. Band quantifications corresponding to Figure S4. Intensity ratio of each band normalized with loading control p84 and the LacZ control cell line (n=3, data presented as the mean ± SD). DK, *POLQ*/*FANCD2* double KO; F, *FANCD2* KO; P, *POLQ* KO; LacZ, negative control of CRISPR KO.

Table S1. List of sgRNA oligos designed for functional knockout of *FANCD2* and *POLQ* using the CRISPR/Cas9 System.

sgRNA Name	Target sequence	Forward primer sequence	Reverse primer sequence
FANCD2_sgRNA2	TACATACTTTGGGATAGGAA	CACCG TACATACTTTGGGATAGGAA	AAAC TTCCTATCCCAAAGTATGTA C
FANCD2_sgRNA3	AGAAGCTCTTTCAGACCCTG	CACCG AGAAGCTCTTTCAGACCCTG	AAAC CAGGGTCTGAAAGAGCTTCT C
POLQ_sgRNA1	TTAGTAAGGATACCCGAACC	CACCG TTAGTAAGGATACCCGAACC	AAAC GGTTCGGGTATCCTTACTAA C
POLQ_sgRNA4	CATGATACGAGCTATTCTGG	CACCG CATGATACGAGCTATTCTGG	AAAC CCAGAATAGCTCGTATCATG C
Negative Control	GTTCCGCGTTACATAACTTA	CACCG GTTCCGCGTTACATAACTTA	AAAC TAAGTTATGTAACGCGGAAC C

Table S2. List of the primers used for Q-PCR.

Gene	Forward	Reverse
IFIT1	TACCTGGACAAGGTGGAGAA	GTGAGGACTGTTGGCTAGA
IFIT2	TGTGCAACCTACTGGCCTAT	TTGCCAGTCCAGAGGTGAAT
IFIT3	CTTCAGTATTTACTTGAGGCAGAC	CTTGGTGACCTCACTCATGAC
IFI6	TCGCTGATGAGCTGGTCTGC	ATTACCTATGACGACGCTGC
ISG15	GCGAACTCATCTTTGCCAGTA	CCAGCATCTTCACCGTCAG
OAS1	CGCCTAGTCAAGCACTGGTA	CAGGAGCTCAGGGCATA
OAS2	TCCAGGGAGTGGCCATAG	TCTGATCCTGGAATTGTTTTAAGTC
MX1	ATGAGCTAATCACCCTGGAG	ATACCCAATGTCAGCAGGC
CCL5	TGCCCACATCAAGGAGTATTT	CTTTCGGGTGACAAAGACG
CXCL10	GGCCATCAAGAATTTACTGAAAGCA	TCTGTGTGGTCCATCCTTGGAA
STING	CATGGGCTGGCATGGTCATA	ATATACAGCCGCTGGCTCAC
TNF-α	CCTCTCTCTAATCAGCCCTCTG	GAGGACCTGGGAGTAGATGAG
IFN-α	GTGAGGAAATACTTCCAAAGAATCAC	TCTCATGATTTCTGCTCTGACAA
IFN-β	AGCTGAAGCAGTTCCAGAAG	AGTCTCATTCCAGCCAGTGC
IFN-γ	GTGGTGCTGGTGACTTTGG	CTCCTGTGGTGACAGAGATTTG
TNFRSF1B	CGGGAGCTCAGATTCTTCCC	GTCTCCAGCTGTGACCGAAA
POLQ	GATTGAGCCAGAGTCTGTTGG	TCCATAAATGATCCCATAGCAA
GAPDH	GGTCTCCTCTGACTTCAACA	GTGAGGGTCTCTCTCTTCCT

Table S3. List of the antibodies used in the study.

Antibody Target	Manufacturer	Catalog No.	Host	Concentration
POLQ	Self-raised	NA	Mouse	1:1000
BRCA2	Calbinchem	OP95-100UGCN	Mouse	1:500
FANCD2	SANTA CRUZ	sc-20022	Mouse	1:1000
p84	GeneTex	GTX70220	Mouse	1:20000
α-Tubulin	GeneTex	GTX112141	Rabbit	1:10000
cGAS	Cell Signaling Technology	15102	Rabbit	1:1000
STING	Cell Signaling Technology	13647	Rabbit	1:1000
Vinculin	Cell Signaling Technology	4650	Rabbit	1:1000
LAMIN A/C	Cell Signaling Technology	4777	Mouse	1:500
Phospho-STAT1 (Tyr701)	Cell Signaling Technology	9167	Rabbit	1:1000

Phospho-H2A.X (Ser139)	Cell Signaling Technology	9718	Rabbit	1:500
Phospho-CHK2 (Thr68)	Cell Signaling Technology	2197	Rabbit	1:1000
